# Effects of extrusion technology on flavor, texture and microstructure of soy-based high-moisture meat analogs and identification of characteristic aroma components

**DOI:** 10.1016/j.fochx.2026.103851

**Published:** 2026-04-09

**Authors:** Shuwei Wang, Tianyu Dong, Jie Sun, Haitao Chen, Huiying Zhang

**Affiliations:** Beijing Key Laboratory of Flavor Chemistry, Beijing Technology and Business University, Beijing 100048, China

**Keywords:** Extrusion temperature, High-moisture extrusion, Moisture content, Molecular sensory science, Soy-based meat analogs

## Abstract

Soy-based high-moisture meat analogs (HMMAs) have been widely applied in plant-based meat products, but the impact of its extrusion process on flavor compounds and the identification of its key flavor components remain unclear. This study systematically investigated the effects of high-moisture extrusion process parameters on the quality of soy-based HMMAs. Results indicated that 70% moisture content and 160 °C extrusion temperature effectively promoted the formation of dense fibrous structure, improved textural properties, and yielded the lowest total volatile off-flavor compounds. SAFE-GC–MS coupled with molecular sensory analysis identified 20 key flavor compounds among 98 volatile compounds. Furanone (FD = 8192) was the major contribution to sweet aroma, while hexanal (OAV = 3144) was the key off-flavor compound. Omission experiments demonstrated that the absence of hexanal and hydroxyacetone significantly altered aroma characteristics (*p* < 0.001). This study provides crucial theoretical basis for optimizing the HMMA process and regulating flavor profiles.

## Highlights

• The optimal extrusion conditions for HMMAs were 70% moisture content and 160 °C.
• A total of 98 volatile compounds were identified in HMMA by SAFE-GC–MS.
• Twenty key aroma compounds were identified through molecular sensory science methods.
• Hexanal and hydroxyacetone exerted significant influence on HMMA flavor.

## 1 Introduction

Soy protein, as the primary and most economical source of high-quality plant protein in plant-based meat analogs (PBMAs), has garnered significant attention (Asgar et al., 2010). United Nations data indicates that since 2000, the global population has grown by nearly 50% and is projected to reach 9.6 billion by 2050 Kołodziejczak et al., 2022. For centuries, humans have heavily relied on meat products as the primary source of high-quality protein in their diets. However, excessive consumption of animal protein leads to increased intake of animal fats and cholesterol, posing potential threats to human health (Zhubi-Bakija et al., 2021). With economic development and rising living standards, trends toward greener food, ecological sustainability, and healthier lifestyles are influencing the dietary habits of some consumers (Zhang et al., 2022). Consequently, to address humanity's protein needs while alleviating resource pressures from traditional livestock farming, research and development of plant-based protein products has expanded significantly. PBMAs have garnered increasing attention. In 2024, the global PBMA market reached $17.1 billion and is projected to grow at a compound annual growth rate (CAGR) of 7.91% through 2026, exceeding $54.8 billion by 2035 (Li et al., 2025). Soy protein isolate (SPI) boasts a protein content of approximately 90% and is rich in amino acids, particularly high-quality essential amino acids. Consequently, it is extensively utilized in various food processing applications. Coupled with its low cost, SPI has long been recognized as one of the preferred raw materials for PBMA production (Yuan, 2024).

High-moisture extrusion technology is currently the most widely used method for producing PBMA. Under the combined effects of pressure, shear force, and high temperature, proteins denature and their original internal molecular structures are disrupted. The protein molecular chains undergo changes, with the screw's shear force aligning them in an orderly, unidirectional arrangement. Upon cooling, they re-crosslink, ultimately forming an organized protein with a fibrous structure similar to meat—known as HMMA (Zahari et al., 2020). High-moisture extrusion represents a relatively novel protein restructuring technique, that HMMA produces with a meat like texture that can be consumed directly. During extrusion, factors such as moisture content, extrusion temperature, screw speed, and feed rate all influence product quality. Moisture content during extrusion not only affects the fiber structure of HMMA but also influences process parameters like pressure and temperature, as well as the rheological properties of the material, thereby impacting other product quality characteristics. Extrusion temperature directly affects material flowability, state, and the extent of reactions between materials within the barrel. It is a key factor influencing protein denaturation and conformation changes, making it crucial for regulating the quality of PBMA. The rotation of the screw promotes uniform mixing of the material with water and propels the material flow. As the screw speed increases, the shear mixing effect on the material within the extrusion chamber gradually intensifies, facilitating the formation of fibrous structures. The feed rate influences the degree of material filling within the extruder, residence time, and die pressure, thereby altering the degree of heating of the materials and influencing the texture, color and water absorption capacity of the products.

One of the unresolved challenges faced by PBMAs, especially soy protein-based PBMAs, is that their flavor (Kowalczewski et al., 2022). “Off-flavor” refers to unpleasant flavors, including perceptible undesirable tastes, odors, and other sensations (Wang et al., 2022). Off-flavors in PBMAs are typically described as “green”, “grassy”, “beany”, “fatty”, and “bitter” (Leonard et al., 2022). Soy protein possesses unpleasant beany and grassy flavors that limit consumer acceptance. The beany flavor stems from multiple volatile compounds generated during soybean growth and processing. Studies have identified over 20 volatile compounds associated with beany flavor (Table 1), among which hexanal is considered the most significant off-flavor compound (Wang et al., 2021). To enhance the sensory acceptability of PBMA, it is necessary to reduce off-flavor formation or mask the off-flavors. Existing research primarily involves blending soy protein with other proteins, such as pea protein (Zhao et al., 2024), wheat gluten (Guo et al., 2020), fava bean protein (Choi et al., 2025), rice bran protein (Zhao et al., 2024), whey protein (Wittek et al., 2021), or incorporating flavoring additives such as thiamine (Milani & Conti, 2023), yeast (Song et al., 2024), mushroom powder (Ketnawa et al., 2023), spices (Yuan et al., 2023), and other food seasonings to enhance the structure or flavor of PBMA. Alternatively, studies have investigated the effects of extrusion conditions on composite raw materials (González-Galeana et al., 2025; Lin et al., 2006; Ye et al., 2025) or other proteins (Barnés-Calle et al., 2024; Gaber et al., 2025; Toldrà et al., 2025). However, research on the impact of extrusion processing on the flavor of soybean HMMA remains limited, and systematic studies identifying key flavor compounds in soybean HMMA are also scarce.

**Table 1:** Main off-flavor odorants and their aromas (Wang et al., 2021).

Compounds	CAS No.	Aroma attributes
(E)-2-hexenal	6728-26-3	leafy
(E)-2-heptenal	18,829–55-5	soap, fat
(E)-2-octenal	2548-87-0	fatty, green, cucumber
(Z)-3-hexenal	6789-80-6	green
pentanal	110–62-3	irritant
hexanal	66–25-1	cut-grass, green
heptanal	111–71-7	dry fish
nonanal	124–19-6	green, fatty
octanal	124–13-0	fatty, pungent
(E)-2-nonenal	18,829–56-6	beany, green
benzaldehyde	100–52-7	almond
(E,E)-2,4-nonadienal	5910-87-2	fatty
(E,E)-2,4-heptadienal	4313-2-4	fatty, fishy
(E,E)-2,4-decadienal	25,152–84-5	spices
2-isopropyl-3-methoxypyrazine	93,905–03-4	pea-like, earthy
acetic acid	64–19-7	sour
2-heptanone	110–43-0	fragrance
1-penten-3-one	1629-58-9	spicy, onion
1-octen-3-one	4312-99-6	green-beany
2-octanone	111–13-7	soapy, floral
2-penthyfuran	3777-69-3	beany off-favors
2-ethylfuran	3208-16-0	beany, earthy, malty, sweet
1-octen-3-ol	3391-86-4	mushroom
1-pentanol	71–41-0	green, wax
1-penten-3-ol	616–25-1	beany, green
hexanol	111–27-3	beany, green
3-methyl-1-butanol	123–51–3	balsamic

Therefore, this study utilized SPI as raw material and investigated the effects of different extrusion techniques on the flavor, texture, and microstructure of HMMA by adjusting moisture content and extrusion temperature during the extrusion process. Volatile compounds in HMMA were identified using SAFE coupled with GC–MS, while key flavor compounds were determined through GC-O combined with AEDA, OAV calculations, reconstitution and omission experiments. The findings provide theoretical support for the preparation and flavor enhancement of HMMA.

## 2 Materials and methods

### 2.1 Materials and reagents

The SPI utilized in this study was purchased from Shansong Biological Products Co., Ltd. (Linyi, China). It was comprised of 91.6% protein and 4.56% moisture. The two components were determined by the methods described in the Chinese national standard (GB-5009.5-2016, 2016, GB-5009.3-2016, 2016). All indicator standard materials were purchased from Macklin (Beijing, China). All chemicals used were of analytical and chromatographic purity.

### 2.2 Sample preparation of HMMAs with different moisture content

The HMMAs were performed using a AHT36-32D twin-screw extruder (Shandong Arrow Machinery Co., Ltd., Jinan, China). The operational parameters of the extruder were set as follows: a screw speed of 420 rpm, screw diameter (D) was 36 mm, length/diameter ratio of the screw was 32:1, and a constant cooling module temperature of 60 °C. The seven independent temperature control zones were set at 70 °C, 80 °C, 120 °C, 140 °C, 150 °C, 160 °C and 140 °C. All other extrusion conditions remained unchanged, with only the moisture content being adjusted. The resulting extrudates with varying moisture contents were labeled HMMA-40%, HMMA-50%, HMMA-60%, HMMA-70% and HMMA-80%. The collected extrudates were stored in a freezer at −18 °C for further analysis.

### 2.3 Sample preparation of HMMAs with different temperature

The moisture content was set to 70%, and other conditions were the same as 2.2. The first six temperatures of the seven independent temperature control zones of the twin-screw extruder were 70 °C, 80 °C, 120 °C, 140 °C, 150 °C, and 160 °C, respectively. The last temperature zone was set to 120 °C, 130 °C, 140 °C, 150 °C, 160 °C, 170 °C, and 180 °C. The resulting extrudates with varying temperatures were labeled HMMA-120 °C, HMMA-130 °C, HMMA-140 °C, HMMA-150 °C, HMMA-160 °C, HMMA-170 °C and HMMA-180 °C. The collected extrudates were stored in a freezer at −18 °C for further analysis.

### 2.4 Scanning electron microscopic (SEM) analysis

The microstructures of the HMMAs were investigated using SU8010 SEM (Hitachi High-Technologies Corp., Tokyo, Japan). Before the measurement, the HMMA samples were sliced (1 × 4 × 4 mm) and freeze drying for 48 h. The samples were glued to the SEM sample table, plated with gold, and observed through the SEM.

### 2.5 Texture profile analysis

The textural properties of the extruded samples were evaluated by texture profile analysis (TPA) using a TMS-Touch (Food Technology Corporation, Virginia, USA). All samples were thawed at 25 °C and cut into a cuboid shape of 10 mm × 40 mm before analysis. Samples were subjected to a double compression test using a 12.7 mm diameter cylindrical probe to compress 60% of its original thickness at a speed of 1 mm/s. The textural properties for hardness (N), springiness, resilience and cohesiveness were then recorded.

### 2.6 Electronic nose (*E*-nose) analysis

The Portable Electronic Nose System (PEN 3, Airsense, Berlin, Germany) contains ten metal oxide semiconductors that provides selectivity for volatile compound classes. Each sample (5 g) was put into 50 mL bottle. Sample were kept in a 25 °C water bath for 20 min to reduce sensor drift caused by environmental changes. Gas was then injected into the detection system through the probe from the headspace of the sample at a flow rate of 300 mL/min. The clean time was 60 s and the data collection time was 180 s, which made the sample data gradually stable. Five parallel tests were conducted for each sample.

### 2.7 Sensory analysis

This study has obtained ethical approval from the Scientific Research Ethics Committee of Beijing Technology and Business University (Audit 2025 No. 151). During the study, agreements protecting the rights and privacy of all participants were signed. All participating panels were informed about the purpose of the sensory evaluation and provided detailed information regarding their privacy and right to withdraw from the study at any time. Each participant signed a consent form agreeing to participate in sensory research and to the use of their information. The sensory evaluation panel consisted of 15 trained judges (6 males and 9 females) recruited from the Beijing Key Laboratory of Flavor Chemistry at Beijing Technology and Business University. All panelists had experience in food sensory evaluation and had undergone training in quantitative descriptive analysis (QDA). Assessment was carried out in the sensory laboratory at a temperature of 25 °C. In the training process, the team members first gave sensory descriptors to describe the aroma of a HMMA, and then recorded statistics on the given descriptors to select the most frequent descriptors as quantitative descriptors. Finally, eight descriptors were obtained, namely, bean-like, beany, sweet, milky, grassy, fatty, cereal, and fermented. During the sniffing period of each sample, assessors were asked to assess intensity on a 9-point intensity scale for each flavor with 1–3 being weak, 4–6 being medium and 7–9 being strong. The averages of the flavor descriptors were plotted on spider web diagrams.

### 2.8 Isolation of aroma compounds by solvent-assisted flavor evaporation (SAFE)

HMMAs and SPI were extracted using dichloromethane. The extract solution was collected after filtration, and 25 μL of 2-methyl-3-heptanone (7.31 mg/mL) was added as the internal standard. The volatile compounds in HMMAs and SPI were isolated using a SAFE apparatus under vacuum. The water bath was maintained at 50 °C, and liquid nitrogen was used to ensure a very low temperature. The extract was dried with anhydrous sodium sulfate (Na_2_SO_4_) and concentrated to approximately 2 to 3 mL using a Vigreux column (50 × 1 cm; Beijing Jingxing Glassware Co., Ltd.) at 45 °C, and then further concentrated to 1 mL under a gentle flow of nitrogen gas. All extraction experiments were repeated three times. The samples were sealed and stored in a − 40 °C refrigerator for subsequent analysis.

### 2.9 Gas chromatography–mass spectrometry (GC–MS) analysis

A Thermo Fisher Trace 1310 gas chromatograph (Thermo Fisher Scientific, Waltham, MA) coupled with a single quadrupole (ISQ) mass spectrometer (Thermo Fisher Scientific) was used to identify and quantify the aroma compounds. Separations were performed using a TG-Wax column (30 m × 0.25 mm i.d., 0.25-μm film thickness; Thermo Fisher Scientific). Helium was used as the carrier gas and delivered to the column at a constant flow rate of 1.0 mL/min. The initial oven temperature was set at 40 °C and held for 3 min, then increased to 160 °C at a rate of 3 °C/min, followed by a ramp to 220 °C at 5 °C/min, which was held for 6 min. The sample was injected in a 1:10 split ratio with an injection volume of 1 μL. The MS conditions were as follows: ionization energy, 70 eV; MS transfer line temperature, 240 °C; ion source temperature, 250 °C; mass range, *m*/*z* 40 to 550; and solvent delay, 5 min.

### 2.10 Gas chromatography–olfactometry (GC-O) analysis and aroma extraction dilution analysis (AEDA)

The GC-O-MS system consisted of a GC–MS instrument equipped with an olfactometric detector (ODP3; Gerstel, Mülheim an der Ruhr, Germany). The column and operating conditions were the same as those used in the GC–MS analysis. The sample was desorbed during injection, separated by the gas chromatograph, and the resulting aroma compounds were directed to the mass spectrometer and the olfactory interface in a 1:1 split ratio. Flavor dilution (FD) factors based on AEDA were determined. The HMMA volatile isolate was stepwise diluted 1:2 with dichloromethane to obtain dilution levels of 1:2, 1:4, 1:8, 1:16, up to 1:8192 of the initial solution, and the resulting series of isolates was analyzed using GC-O-MS under the same conditions described above. Each dilution level was evaluated by three professionally trained panelists, and an odor was identified as an aroma-active compound only if it was detected by at least two of the panelists.

### 2.11 Identification and quantitation of volatile compounds

The aroma compounds in HMMAs were identified by comparing their mass spectra, retention indices (RIs), and odor qualities with data from the NIST 20 mass spectral database and with those of standard reference compounds. RIs were calculated for each volatile compound using the retention times of a homologous series of C_6_–C_28_ n-alkanes.

The aroma compounds in HMMAs identified by GC-O were quantitated by constructing standard curves using 2-methyl-3-heptanone as the internal standard. MS was conducted in single-ion monitoring (SIM) mode. The standard curves were established by plotting the response factors of the standard compounds and the 2-methyl-3-heptanone internal standard against their concentration ratios.

### 2.12 Calculation of odor activity values (OAVs)

The OAVs were determined to assess the contributions of volatile compounds to the aroma profiles of HMMAs. The OAVs of the aroma compounds were calculated based on the ratio of their concentrations detected in HMMAs to their respective aroma thresholds in water. It is generally accepted that an OAV ≥ 1 indicates a significant contribution of a compound to the overall flavor of a sample.

### 2.13 Recombination and omission experiments

The aroma compounds used to construct the recombination model were identified based on the results of GC-O analysis. All aroma-active compounds were mixed into a HMMA mock matrix (water) at their detected concentrations. Both the recombination model and HMMA samples were subjected to sensory evaluation using a standardized sensory analysis method.

Descriptive sensory evaluation panel assessed all aroma missing models using a triangle test, and missing models were prepared by removing one compound from the complete composite system. The sensory panel of the omission experiments were the same as that for the sensory analysis.

### 2.14 Statistical analysis

Experimental data were collected and organized using Excel (Microsoft Office 2016, Redmond, WA, USA). The results of the experiments were expressed as the mean of three replicates ± standard deviation. Aroma profiles, bar charts, line graphs and principal component analysis (PCA) were plotted using Origin 2025b (OriginLab Corporation, Northampton, MA, USA). IBM SPSS Statistics 24 was employed to perform one-way analysis of variance (ANOVA) with Duncan's multiple range test (*P* < 0.05) for significance assessment. TBtools v2.0 was used to construct hierarchical clustering heatmaps.

## 3 Results and discussion

### 3.1 Effect of moisture content on HMMAs

#### 3.1.1 Effect of moisture content on aroma and sensory profiles of HMMAs

**Fig. 1:**
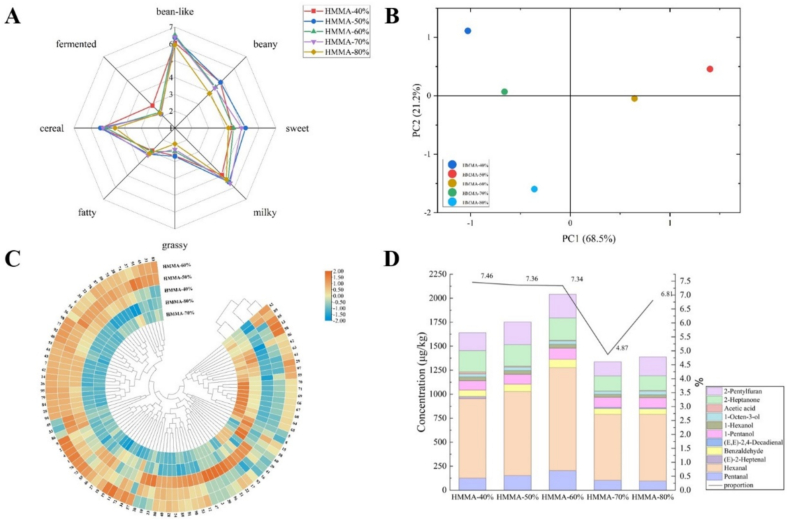
The aroma profiles and compound composition of HMMAs at different moisture contents. (A) The aroma profiles of HMMAs. (B) PCA chart for aroma profiles of HMMAs. (C) Heatmap analysis of aroma compounds in HMMAs. (D) The content and proportion of off-flavor compounds in HMMAs. The numbers correspond to the compound No. in Table 2.

To understand the effect of moisture content on the aromatic characteristics of HMMAs, QDA was performed, and the results are shown in Fig. 1 A. The aromatic characteristics of HMMAs were described using eight attributes: bean-like, beany, sweet, milky, grassy, fatty, cereal, and fermented aroma. As moisture content increased, the intensity of bean-like, beany, sweet, grass, cereal, milky and fatty aroma followed a pattern of initially increasing and then decreasing. Among these, the beany, sweet, cereal and grass aroma were strongest at HMMA-50%, while the aromas were weakest at HMMA-80%. Milky and fatty aroma were weakest at HMMA-40% and strongest at HMMA-70%. HMMA-70% exhibits stronger sweet, milky and fatty aroma, along with weaker grass and fermented aroma, resulting in a more balanced overall aroma.

The *E*-nose can obtain all information related to volatile compounds in a sample without separating the volatiles, expressing the overall aroma profile presented by the volatile compounds to the sample (Zhu et al., 2017). In order to better display the aroma differences of HMMAs at different moisture contents, PCA was employed for the statistical analysis of the E-nose results. As shown in Fig. 1 B, the first principal component (PC1) comprised 68.5% of the total variable information, and the second principal component (PC2) comprised 21.2%. The total contribution rate of the two principal components was 89.7%, indicating that the analysis results can reflect the main characteristic information of the sample. HMMA-50% and HMMA-80% were located in the first and third quadrants, respectively, and were positioned at a considerable distance from other sample points. This indicates that the aromas of these two samples exhibit significant differences compared to other HMMAs. Consequently, the overall aroma differences revealed by the E-nose PCA analysis corroborate the variations in specific attribute intensities obtained from sensory evaluation, collectively demonstrating the significant influence of moisture content on the HMMA aroma profile.

As the results of the application of SAFE on HMMAs prepared at different moisture content, the volatile compounds are shown in Table 2. A total of 98 volatile compounds were identified and divided into 9 chemical classes, including 8 aldehydes, 14 alcohols, 8 acids, 11 esters, 11 ketones, 19 hydrocarbons, 22 heterocyclic compounds, 3 phenols, and 2 others. Among these, 80 compounds were detected in all 5 samples. It was indicated that moisture content has a significant impact on the aroma of HMMAs. As moisture content increased, the total amount of volatile compounds in HMMAs first increased and then decreased, with the highest content in HMMA-60% (27,812.64 ± 2293.44 μg/kg) and the lowest in HMMA-80% (20,406.59 ± 971.9 μg/kg). Research has shown that most food aroma compounds exhibited high activity in aqueous solutions, resulting in higher relative volatility (Guo et al., 2020). As the moisture content of the raw material increased, moisture evaporated from HMMA, leading to greater loss of more volatile flavor compounds. The content of aldehydes was highest at a moisture content of 60% (1954.44 μg/kg), with the primary aldehyde compound, hexanal, also reached its peak at 60%. Hexanal serves as a key marker for lipid oxidation, suggesting that moderate moisture content may have promoted lipid oxidative degradation, generating substantial amounts of aldehydes imparting grassy and fatty flavors. Ketones had a high and stable content between 40% and 60% moisture content, then decrease significantly by approximately 50% at 70% and 80%. Among them, acetoin, hydroxyacetone, and the off-flavor compound 2-heptanone all showed this trend. In HMMA, maltol is the most abundant compound, accounting for 24.23% to 36.38% of the total compound content. Maltol has a caramel aroma, which may be one of the reasons why HMMA has a strong sweet aroma. Furthermore, studies have indicated that 1-octen-3-ol, benzyl alcohol, hexanal, and benzaldehyde were detected in textured vegetable protein (TVP) produced from peas, wheat, and peanuts respectively. However, (E,*E*)-2,4-decadienal and phenylethyl alcohol were exclusively present in textured peanut protein, while 2,3-butanediol was found only in textured pea protein (Pu et al., 2024). To visually distinguish changes in volatile compounds across HMMA samples with varying moisture content and elucidate the effect of moisture content on HMMA aroma compounds, heatmap hierarchical cluster analysis (HCA) was employed to visualize relative volatile compounds. The color gradient from blue to red reflects increasing compound abundance. As shown in Fig. 1 C, relative volatile compound concentrations differ significantly among samples. Row clustering was a bottom-up process (Verbeek et al., 2018). HMMA-70% and HMMA-80% clustered into one class, while the other three samples gradually clustered into another class. This indicated that HMMA exhibits certain similarities in aroma compounds when moisture content ranges from 40% to 60% and from 70% to 80%, respectively. However, significant differences exist between these two classes.

**Table 2:** Changes in the content of volatile compounds in HMMAs during the extrusion process at different moisture contents.

Type	No.	CAS	Compounds	RI	Concentration (μg/kg)					Identification
					HMMA-40%	HMMA-50%	HMMA-60%	HMMA-70%	HMMA-80%	
Aldehydes	1	110–62-3	Pentanal	/	126.22 ± 10.55 ^c^	152.98 ± 3.99 ^b^	204.51 ± 5.99 ^a^	103.28 ± 5.78 ^d^	96.61 ± 2.66 ^d^	MS
	2	66–25-1	Hexanal	1081	827.96 ± 48.38 ^b^	874.99 ± 27.77 ^b^	1072.32 ± 35.13 ^a^	687.63 ± 7.23 ^c^	694.12 ± 13.18 ^c^	MS, RI, S
	3	18,829–55-5	(E)-2-Heptenal	1317	23.25 ± 0.78 ^a^	–	–	–	–	MS, RI
	4	100–52-7	Benzaldehyde	1506	65.67 ± 2.1 ^c^	76.93 ± 6.87 ^b^	87.26 ± 9.13 ^a^	61.32 ± 1.74 ^c^	57.71 ± 3.43 ^c^	MS, RI, S
	5	25,152–84-5	(E,E)-2,4-Decadienal	1796	–	–	–	10.2 ± 0.6 ^a^	9.88 ± 1.03 ^a^	MS, RI, S
	6	121–33-5	Vanillin	2550	410.96 ± 14.58 ^a^	428.51 ± 35.26 ^a^	389.23 ± 56.41 ^a^	304.94 ± 20.48 ^b^	318.61 ± 6.78 ^b^	MS, RI, S
	7	134–96-3	4-Hydroxy-3,5-dimethoxybenzaldehyde	/	161.92 ± 16.79 ^a^	160.26 ± 19.21 ^a^	162.42 ± 26.07 ^a^	108.85 ± 9.65 ^b^	114.62 ± 11 ^b^	MS
	8	123–08-0	4-Hydroxybenzaldehyde	/	48.46 ± 9.66 ^cd^	53.61 ± 4.22 ^bc^	38.7 ± 7.35 ^d^	64.03 ± 0.41 ^b^	82.91 ± 7.9 ^a^	MS
Subtotal					1664.44 ± 102.84	1747.28 ± 97.32	1954.44 ± 140.08	1340.25 ± 45.89	1374.46 ± 45.98	
Alcohols	9	75–85-4	Amylene hydrate	1015	437.13 ± 37.3 ^ab^	443.12 ± 25.41 ^ab^	473.69 ± 12.98 ^a^	430.41 ± 29.22 ^ab^	420.1 ± 19.3 ^b^	MS, RI, S
	10	71–36-3	1-Butanol	1144	6.31 ± 0.49 ^a^	4.43 ± 0.16 ^b^	2.3 ± 0.21 ^d^	2.96 ± 0.32 ^c^	2.12 ± 0.11 ^d^	MS, RI, S
	11	1569-50-2	3-Penten-2-ol	1169	195.34 ± 36.53 ^b^	204.9 ± 7.91 ^b^	336.12 ± 5.8 ^a^	153.67 ± 9.63 ^c^	203.31 ± 4.43 ^b^	MS, RI, S
	12	623–37-0	3-Hexanol	1194	9.29 ± 0.83 ^a^	7.76 ± 0.4 ^b^	8.72 ± 0.09 ^a^	6.99 ± 0.38 ^bc^	6.31 ± 0.15 ^c^	MS, RI, S
	13	71–41-0	1-Pentanol	1247	98.42 ± 10.38 ^b^	102.23 ± 3.69 ^b^	115.74 ± 2.56 ^a^	105.2 ± 1.2 ^ab^	105.17 ± 6.56 ^ab^	MS, RI, S
	14	111–27-3	1-Hexanol	1348	39.3 ± 2.22 ^a^	39.55 ± 1.34 ^a^	40.64 ± 1.4 ^a^	31.88 ± 1.55 ^b^	31.44 ± 0.35 ^b^	MS, RI, S
	15	3391-86-4	1-Octen-3-ol	1444	31.65 ± 1.54 ^b^	36.66 ± 3.11 ^a^	35.34 ± 0.15 ^a^	31.16 ± 0.29 ^b^	37.09 ± 0.25 ^a^	MS, RI, S
	16	104–76-7	2-Ethylhexanol	1481	23.53 ± 1.11 ^a^	14.68 ± 1.17 ^c^	14.54 ± 0.33 ^c^	20.36 ± 1.12 ^b^	19.12 ± 0.25 ^b^	MS, RI, S
	17	513–85-9	2,3-Butanediol	1574	18.45 ± 0.71 ^a^	14.79 ± 1.18 ^c^	16.93 ± 0.41 ^b^	14.36 ± 0.3 ^c^	16.06 ± 0.1 ^b^	MS, RI, S
	18	112–34-5	Diethylene glycol monobutyl ether	1778	14.16 ± 0.35 ^a^	13.95 ± 0.69 ^a^	14.32 ± 2.78 ^a^	12.5 ± 1.33 ^a^	13.9 ± 0.65 ^a^	MS, RI, S
	19	100–51-6	Benzyl alcohol	1859	38.91 ± 0.63 ^b^	41.97 ± 0.18 ^b^	46.46 ± 2.55 ^a^	38.74 ± 3.03 ^b^	41.67 ± 0.74 ^b^	MS, RI, S
	20	60–12-8	Phenylethyl alcohol	1891	28.17 ± 1.63 ^a^	24.25 ± 4.31 ^a^	14.5 ± 1.4 ^b^	13.55 ± 1.41 ^b^	13.64 ± 0.84 ^b^	MS, RI, S
	21	122–99-6	2-Phenoxyethanol	2124	101.62 ± 3.84 ^a^	41.58 ± 0.39 ^c^	52.13 ± 5.53 ^b^	48.38 ± 3.22 ^b^	29.19 ± 1.18 ^d^	MS, RI, S
	22	36,653–82-4	1-Hexadecanol	2363	19.08 ± 0.47 ^b^	23.57 ± 1.08 ^a^	15.16 ± 2.62 ^c^	9.61 ± 1.63 ^d^	–	MS, RI, S
Subtotal					1061.36 ± 98.03	1013.44 ± 51.02	1186.59 ± 38.81	919.77 ± 54.63	939.12 ± 34.91	
Acids	23	64–19-7	Acetic acid	1452	20.25 ± 3.71 ^a^	8.95 ± 0.18 ^b^	6.39 ± 0.39 ^b^	2.58 ± 0.31 ^c^	7.41 ± 0.45 ^b^	MS, RI, S
	24	109–52-4	Pentanoic acid	1731	5.94 ± 0.65 ^a^	5.17 ± 0.29 ^a^	5.15 ± 0.72 ^a^	2.89 ± 0.19 ^b^	3.01 ± 0.2 ^b^	MS, RI, S
	25	142–62-1	Hexanoic acid	1833	210.53 ± 8.91 ^a^	223.3 ± 13.85 ^a^	229.69 ± 2.89 ^a^	133.4 ± 7.37 ^b^	142.02 ± 13.56 ^b^	MS, RI, S
	26	124–07-2	Octanoic acid	2046	109.15 ± 2.43 ^a^	110.39 ± 2.57 ^a^	113.14 ± 12.11 ^a^	81.13 ± 6.18 ^b^	76.01 ± 1.56 ^b^	MS, RI, S
	27	112–05-0	Nonanoic acid	2153	30.46 ± 1.67 ^a^	28.71 ± 1.13 ^a^	28.14 ± 4.07 ^a^	22.1 ± 2.48 ^b^	26.23 ± 0.35 ^ab^	MS, RI, S
	28	1871-67-6	(E)-2-Octenoicacid	2173	16.37 ± 1.13 ^ab^	19.12 ± 1.08 ^a^	18.69 ± 3.08 ^a^	14.13 ± 0.77 ^b^	–	MS, RI, S
	29	334–48-5	Decanoic acid	2258	11.81 ± 0.49 ^c^	12.15 ± 2.1 ^bc^	16.02 ± 1.14 ^a^	14.54 ± 1.4 ^ab^	14.8 ± 1.16 ^a^	MS, RI, S
	30	57–10-3	Hexadecanoic acid	/	684.72 ± 97.68 ^a^	–	–	–	–	MS
Subtotal					1089.23 ± 116.67	407.79 ± 21.2	417.22 ± 24.4	270.77 ± 18.7	269.48 ± 17.28	
Esters	31	96–48-0	γ-Butyrolactone	1614	–	55.44 ± 1.32 ^a^	46.63 ± 5.15 ^b^	18.37 ± 0.43 ^c^	3.69 ± 0.24 ^d^	MS, RI, S
	32	695–06-7	5-Ethyldihydro-2(3H)-furanone	1686	17.11 ± 1.31 ^a^	13.38 ± 0.03 ^c^	15.46 ± 1.06 ^b^	12.24 ± 0.63 ^c^	13.39 ± 0.15 ^c^	MS, RI, S
	33	923–26-2	2-Hydroxypropyl methacrylate	1724	19.72 ± 0.78 ^b^	23.68 ± 0.95 ^a^	7.37 ± 0.96 ^d^	10.37 ± 0.63 ^c^	10.6 ± 0.32 ^c^	MS, RI, S
	34	104–50-7	γ-Octanoic lactone	1894	8.14 ± 0.14 ^bc^	9.14 ± 0.83 ^ab^	10.1 ± 1.59 ^a^	7.06 ± 0.66 ^c^	7.92 ± 0.35 ^bc^	MS, RI
	35	104–61-0	γ-Nonanolactone	2005	23.14 ± 1.03 ^a^	23.82 ± 0.9 ^a^	22.04 ± 1.89 ^a^	17.91 ± 1.67 ^b^	17.95 ± 0.83 ^b^	MS, RI, S
	36	112–39-0	Hexadecanoic acid, methyl ester	2195	28.46 ± 1.08 ^b^	39.94 ± 1.76 ^a^	23.23 ± 3.69 ^c^	12.68 ± 1.53 ^d^	11.97 ± 1.64 ^d^	MS, RI, S
	37	628–97-7	Hexadecanoic acid, ethyl ester	2236	17.44 ± 1.11 ^c^	42.66 ± 2.92 ^a^	23.66 ± 1.92 ^b^	12.56 ± 2.18 ^d^	11.31 ± 2.07 ^d^	MS, RI, S
	38	131–11–3	Dimethyl phthalate	2277	25.18 ± 0.3 ^b^	27.6 ± 1.65 ^b^	28.57 ± 4.57 ^b^	24.86 ± 2.67 ^b^	35.12 ± 0.79 ^a^	MS, RI, S
	39	84–69-5	Diisobutyl phthalate	2520	53.79 ± 9.97 ^a^	39.27 ± 6.25 ^bc^	32.75 ± 4.85 ^c^	46.54 ± 3.23 ^ab^	32.6 ± 5.44 ^c^	MS, RI, S
	40	5469-16-9	(+/−)-3-hydroxy-γ-butyrolactone	2592	557.73 ± 17.77 ^a^	506.39 ± 68.75 ^a^	418.71 ± 17.1 ^b^	38.26 ± 2.31 ^c^	47.72 ± 4.51 ^c^	MS, RI, S
	41	84–74-2	Dibutyl phthalate	2676	330.61 ± 42.47 ^a^	260.53 ± 34.99 ^bc^	217.15 ± 34.17 ^cd^	292.95 ± 36.19 ^ab^	174.36 ± 34.59 ^d^	MS, RI, S
Subtotal					1081.32 ± 75.96	1041.85 ± 120.35	845.67 ± 76.95	493.8 ± 52.13	366.63 ± 50.93	
Ketones	42	110–43-0	2-Heptanone	1179	221.29 ± 11.64 ^a^	224.61 ± 8.09 ^a^	232.65 ± 10.05 ^a^	156.99 ± 7.68 ^b^	151.94 ± 3.33 ^b^	MS, RI, S
	43	513–86-0	Acetoin	1275	167.89 ± 11.11 ^a^	170.34 ± 2.44 ^a^	171.53 ± 4.83 ^a^	112.66 ± 1.6 ^b^	111.38 ± 1.44 ^b^	MS, RI, S
	44	116–09-6	Hydroxyacetone	1289	733.44 ± 48.59 ^a^	765 ± 4.17 ^a^	740.53 ± 25.28 ^a^	310.65 ± 3.87 ^b^	298.94 ± 11.26 ^b^	MS, RI, S
	45	1120-73-6	2-methylcyclopentenone	1359	3.00 ± 0.28 ^b^	3.65 ± 0.13 ^a^	3.57 ± 0.15 ^a^	2.82 ± 0.38 ^b^	2.71 ± 0.25 ^b^	MS, RI, S
	46	5077-67-8	1-Hydroxy-2-butanone	1366	8.06 ± 0.06 ^a^	7.59 ± 0.29 ^a^	8.01 ± 0.4 ^a^	5.23 ± 0.33 ^c^	6.14 ± 0.19 ^b^	MS, RI, S
	47	592–20-1	Acetoxyacetone	1465	4.20 ± 0.27 ^a^	–	–	–	–	MS, RI, S
	48	30,086–02-3	3,5-Octadien-2-one	1561	24.66 ± 0.76 ^b^	27.99 ± 1.37 ^a^	28.99 ± 0.9 ^a^	20.28 ± 0.65 ^c^	21.74 ± 0.98 ^c^	MS, RI, S
	49	98–86-2	Acetophenone	1632	1.60 ± 0.04 ^b^	2.11 ± 0.21 ^a^	–	–	–	MS, RI, S
	50	80–71-7	Methyl cyclopentenolone	1816	7.24 ± 0.27 ^b^	8.44 ± 0.1 ^a^	7.41 ± 0.67 ^b^	5.3 ± 0.21 ^c^	3.71 ± 0.34 ^d^	MS, RI, S
	51	21,835–01-8	3-Ethyl-2-hydroxy-2-cyclopenten-1-one	1879	11.94 ± 0.13 ^ab^	12.8 ± 0.33 ^a^	12.55 ± 1.01 ^a^	10.17 ± 0.96 ^c^	10.99 ± 0.3 ^bc^	MS, RI, S
	52	498–02-2	Apocynin	2626	10.36 ± 0.61 ^b^	14.71 ± 2.75 ^a^	15.62 ± 2.46 ^a^	13.14 ± 1.75 ^ab^	12.61 ± 0.51 ^ab^	MS, RI, S
Subtotal					1193.68 ± 73.76	1237.24 ± 19.88	1220.86 ± 45.75	637.24 ± 17.43	620.16 ± 18.6	
Hydrocarbons	53	15,869–93-9	3,5-Dimethyloctane	1042	39.58 ± 2.99 ^a^	32.62 ± 0.19 ^b^	33.94 ± 1.53 ^b^	24.41 ± 3.11 ^c^	22.87 ± 3.67 ^c^	MS
	54	6975-98-0	2-Methyldecane	1061	18.06 ± 3.48 ^b^	25.86 ± 0.07 ^a^	19.94 ± 2.49 ^b^	–	–	MS
	55	1120-21-4	Undecane	1100	65.26 ± 12.79 ^ab^	55.57 ± 1.73 ^bc^	67.82 ± 2.52 ^a^	47.91 ± 1.63 ^c^	45.74 ± 1.2 ^c^	MS, RI, S
	56	100–41-4	Ethylbenzene	1119	23.55 ± 3.15 ^b^	27.77 ± 0.61 ^a^	27.52 ± 0.96 ^a^	28.15 ± 0.49 ^a^	28.67 ± 2.21 ^a^	MS, RI, S
	57	112–40-3	Dodecane	1199	11.01 ± 1.93 ^abc^	9.29 ± 0.39 ^bc^	12.31 ± 1.86 ^a^	8.96 ± 0.39 ^c^	11.55 ± 0.25 ^ab^	MS, RI, S
	58	100–42-5	Styrene	1247	98.42 ± 10.38 ^b^	102.23 ± 3.69 ^b^	115.74 ± 2.56 ^a^	105.2 ± 1.2 ^ab^	105.17 ± 6.56 ^ab^	MS, RI, S
	59	544–76-3	Hexadecane	1594	4.48 ± 0.47 ^b^	6.5 ± 1.17 ^a^	8.03 ± 1.12 ^a^	8.18 ± 1.07 ^a^	6.72 ± 0.56 ^a^	MS, RI, S
	60	629–78-7	Heptadecane	1697	–	–	–	–	21.22 ± 2.78 ^a^	MS, RI, S
	61	593–45-3	Octacosane	1793	–	–	11.35 ± 2.24 ^a^	13.26 ± 1.93 ^a^	13.06 ± 2.06 ^a^	MS, RI, S
	62	629–92-5	Nonadecane	1889	21.39 ± 3.84 ^d^	48.95 ± 8.12 ^c^	80.55 ± 12.42 ^b^	90.73 ± 10.99 ^ab^	104.96 ± 20.06 ^a^	MS, RI, S
	63	112–95-8	Eicosane	1989	147.34 ± 7.63 ^c^	214.1 ± 34.84 ^c^	311.68 ± 38.89 ^b^	307.62 ± 51.56 ^b^	411.96 ± 63.38 ^a^	MS, RI, S
	64	629–94-7	Heneicosane	2090	281.53 ± 4.51 ^c^	336.64 ± 58.92 ^c^	637.34 ± 126.29 ^b^	894.53 ± 103.54 ^a^	659.48 ± 52.92 ^b^	MS, RI, S
	65	629–97-0	Docosane	2189	344.58 ± 21.63 ^c^	431.44 ± 35.62 ^c^	827.65 ± 129.13 ^b^	1285.67 ± 76.69 ^a^	811.6 ± 11.48 ^b^	MS, RI, S
	66	638–67-5	Tricosane	2289	387.59 ± 51.22 ^d^	525.42 ± 19.6 ^c^	977.11 ± 139.65 ^b^	1594.11 ± 61.75 ^a^	908.82 ± 39.86 ^b^	MS, RI, S
	67	646–31-1	Tetracosane	2389	381.54 ± 71.5 ^d^	614.91 ± 6.26 ^c^	1114.63 ± 149.35 ^b^	1875.7 ± 60.15 ^a^	1013.91 ± 74.58 ^b^	MS, RI, S
	68	629–99-2	Pentacosane	2487	410 ± 69.35 ^d^	682.45 ± 17.72 ^c^	1257.29 ± 165.8 ^b^	2144.59 ± 93.19 ^a^	1131.09 ± 98.02 ^b^	MS, RI, S
	69	630–01–3	Hexacosane	2585	339.53 ± 60.96 ^d^	693.92 ± 41.54 ^c^	1338.77 ± 208.56 ^b^	2387.91 ± 124.95 ^a^	1332.55 ± 92.71 ^b^	MS, RI, S
	70	593–49-7	Heptacosane	2685	329.44 ± 46.52 ^d^	674.63 ± 58.62 ^c^	1189.31 ± 150.05 ^b^	2137.51 ± 162.08 ^a^	1082.16 ± 89.71 ^b^	MS, RI, S
	71	630–02-4	Octacosane	2785	263.63 ± 46.89 ^d^	591.31 ± 59.02 ^c^	1018.07 ± 121.18 ^b^	1854.45 ± 163.38 ^a^	922.31 ± 67.12 ^b^	MS, RI, S
Subtotal					3166.93 ± 419.24	5073.61 ± 348.11	9049.05 ± 1256.6	14,808.89 ± 918.1	8633.84 ± 629.13	
heterocycles	72	3777-69-3	2-Pentylfuran	1222	186.48 ± 7.69 ^b^	235.48 ± 14.39 ^a^	246.29 ± 10.41 ^a^	146.9 ± 5.26 ^c^	197.47 ± 4.88 ^b^	MS, RI, S
	73	98–01-1	Furfural	1458	14.67 ± 1.72 ^b^	10.23 ± 0.69 ^c^	17.38 ± 1.16 ^a^	7.91 ± 0.43 ^d^	8.72 ± 0.36 ^cd^	MS, RI, S
	74	1192-62-7	2-Acetylfuran	1495	9.48 ± 0.25 ^b^	13.19 ± 1.16 ^a^	10.73 ± 0.27 ^b^	10.25 ± 0.83 ^b^	–	MS, RI, S
	75	98–00-0	2-Furanmethanol	1653	2507.05 ± 107.94 ^b^	2728.31 ± 61.03 ^a^	2453.18 ± 68.96 ^b^	1068.99 ± 21.56 ^c^	418.34 ± 6.01 ^d^	MS, RI, S
	76	3857-25-8	(5-Methyl-2-furyl)methanol	1715	12.73 ± 1.13 ^c^	15.4 ± 1.3 ^b^	18.66 ± 0.23 ^a^	12.44 ± 1.51 ^c^	7.92 ± 0.32 ^d^	MS, RI, S
	77	497–23-4	2(5H)-Furanone	1736	272.66 ± 11.69 ^c^	317.38 ± 5.66 ^b^	352.74 ± 9.3 ^a^	156.43 ± 4.8 ^d^	166.42 ± 3 ^d^	MS, RI, S
	78	636–72-6	2-Thiophenemethanol	1925	9.3 ± 0.43 ^b^	13.72 ± 0.75 ^a^	12.93 ± 0.77 ^a^	4.66 ± 0.74 ^c^	–	MS, RI, S
	79	118–71-8	Maltol	1951	8005.66 ± 317.42 ^a^	8497.26 ± 181.15 ^a^	8477.39 ± 487.08 ^a^	6647.81 ± 363.12 ^b^	6559.75 ± 121.58 ^b^	MS, RI, S
	80	17,678–19-2	2-Furylhydroxymethylketone	1981	76.28 ± 4.44 ^a^	69.52 ± 1.43 ^b^	66.41 ± 5.15 ^b^	34.39 ± 0.14 ^c^	27.21 ± 1.19 ^d^	MS, RI, S
	81	3658-77-3	Furaneol	2021	42 ± 4.18 ^a^	35.48 ± 1.17 ^b^	31.24 ± 3.78 ^b^	23.35 ± 1.06 ^c^	17.23 ± 1.6 ^d^	MS, RI, S
	82	19,322–27-1	4-Hydroxy-5-methyl-3-furanone	2105	22.49 ± 3.87 ^a^	18.45 ± 1.77 ^ab^	19.78 ± 1.46 ^b^	–	–	MS, RI, S
	83	28,564–83-2	2,3-Dihydro-3,5-dihydroxy-6-methyl-4(H)-pyran-4-one	2249	212.3 ± 3.04 ^c^	491.07 ± 34.49 ^a^	498.59 ± 34.89 ^a^	266.69 ± 20.53 ^b^	245.06 ± 13.24 ^bc^	MS, RI, S
	84	496–16-2	2,3-Dihydrobenzofuran	2380	91.68 ± 3.02 ^a^	96.68 ± 3.15 ^a^	96.69 ± 7.14 ^a^	68.89 ± 4.41 ^b^	70.89 ± 1.66 ^b^	MS
	85	32,780–06-6	(*S*)-5-Hydroxymethyldihydrofuran-2-one	2469	48.95 ± 3.26 ^cd^	44.88 ± 1.4 ^d^	65.27 ± 5.79 ^a^	58.87 ± 2.87 ^ab^	52.73 ± 2.86 ^bc^	MS, RI, S
	86	67–47-0	5-Hydroxymethylfurfural	2492	46.85 ± 1.52 ^a^	48.74 ± 3.13 ^a^	14.3 ± 2.26 ^b^	–	–	MS, RI, S
	87	290–37–9	Pyrazine	1206	23.47 ± 0.59 ^b^	29.65 ± 1.05 ^a^	28.33 ± 1.42 ^a^	15.08 ± 0.45 ^c^	8.09 ± 0.7 ^d^	MS, RI, S
	88	109–08-0	2-Methylpyrazine	1259	27.64 ± 1.34 ^a^	20.4 ± 1.73 ^b^	18.26 ± 1.08 ^c^	8.32 ± 0.93 ^d^	–	MS, RI, S
	89	123–32-0	2,5-Dimethyl pyrazine	1322	–	18.53 ± 0.86 ^a^	15.55 ± 0.39 ^b^	12.56 ± 0.62 ^c^	9.55 ± 0.74 ^d^	MS, RI, S
	90	872–50-4	N-Methyl-2-pyrrolidone	1668	363.04 ± 57.58 ^a^	247.38 ± 4.5 ^b^	265.19 ± 11.99 ^b^	129.52 ± 6.3 ^c^	131.22 ± 1.99 ^c^	MS, RI, S
	91	616–45-5	2-Pyrrolidinone	2027	20.85 ± 1.48 ^b^	24.68 ± 0.57 ^a^	22.99 ± 2.99 ^ab^	20.1 ± 1.22 ^b^	20.59 ± 0.54 ^b^	MS, RI, S
	92	120–72-9	Indole	2417	52.64 ± 2.59 ^a^	44.03 ± 0.89 ^b^	48.71 ± 3.71 ^ab^	50.87 ± 7.26 ^ab^	47.18 ± 0.76 ^ab^	MS, RI, S
	93	59–48-3	Oxindole	/	101.35 ± 3.86 ^ab^	115.44 ± 15.91 ^a^	123.84 ± 24.06 ^a^	77.95 ± 10.03 ^b^	74.63 ± 8.33 ^b^	MS, RI, S
Subtotal					12,147.57 ± 539.04	13,135.9 ± 338.18	12,904.45 ± 684.29	8821.98 ± 454.07	8063 ± 169.76	
Phenols	94	106–44-5	p-Cresol	2068	18.61 ± 0.61 ^a^	20.11 ± 1.45 ^a^	20.08 ± 0.74 ^a^	13.02 ± 0.42 ^b^	14.16 ± 1.06 ^b^	MS, RI, S
	95	7786-61-0	2-Methoxy-4-vinylphenol	2179	73.69 ± 3.53 ^b^	84.04 ± 4.61 ^a^	85.98 ± 4.96 ^a^	51.68 ± 3.58 ^c^	54.94 ± 0.41 ^c^	MS, RI, S
	96	96–76-4	2,4-Di-tert-butylphenol	2297	487.42 ± 60.68 ^a^	37.82 ± 1.07 ^c^	102.47 ± 19.4 ^b^	55.13 ± 9.67 ^bc^	50.85 ± 2.91 ^bc^	MS, RI, S
Subtotal					579.72 ± 64.82	141.97 ± 7.13	208.53 ± 25.1	119.83 ± 13.67	119.95 ± 4.38	
Others	97	60–35-5	Acetamide	1771	–	–	4.25 ± 0.26 ^a^	3.78 ± 0.25 ^b^	3.24 ± 0.24 ^c^	MS, RI, S
	98	541–46-8	Isovaleramide	1912	19.54 ± 1.86 ^a^	20.66 ± 0.45 ^a^	21.58 ± 1.2 ^a^	16.62 ± 1.5 ^b^	16.71 ± 0.69 ^b^	MS, RI, S
Subtotal					19.54 ± 1.86	20.66 ± 0.45	25.83 ± 1.46	20.4 ± 1.75	19.95 ± 0.93	
Total					22,003.79 ± 1492.22	23,819.74 ± 1003.64	27,812.64 ± 2293.44	27,432.93 ± 1576.37	20,406.59 ± 971.9	

-, Not detected. Note: Different letters in each row indicate significance (*p* < 0.05).

Eleven off-flavor compounds were identified in HMMAs with varying moisture contents, including pentanal, hexanal, (E)-heptenal, benzaldehyde, (E,E)-2,4-decadienal, 1-pentanol, 1-hexanol, 1-octen-3-ol, acetic acid, 2-heptanone, and 2-pentylfuran, with their concentrations shown in Fig. 1 D. It can be seen that hexanal is the most abundant off-flavor compound, and in HMMA, hexanal is also the third most abundant compound (Table 2), indicating that off-flavor compounds have a significant impact on the flavor of HMMA. At HMMA-70%, the total content of off-flavor compounds is the lowest, and the proportion of off-flavor compounds in the total volatile compound content is also the lowest, which is consistent with the sensory evaluation results shown in the Fig. 1 A.

#### 3.1.2 Effect of moisture content on texture analysis of HMMAs

Texture is a critical characteristic of meat analogs, significantly influencing their perceived viscosity and overall palatability (Tang et al., 2025). A texture analyzer was used to assess the effects of moisture content on the texture properties of HMMA, including hardness, adhesiveness, springiness, gumminess and chewiness. During extrusion, moisture functions as a lubricant and plasticizer, participating in protein stretching and orientation. However, excessively high or low moisture levels impair the fibrous structure of the sample (Dong, 2023; Verbeek et al., 2018). As shown in Table S1, as moisture content increased, the hardness, chewiness, and adhesiveness of HMMAs gradually decreased. Similar results were observed in high-moisture extrudates of pea protein (Sun et al., 2023), hemp seed protein concentrate (Zahari et al., 2023), and soy protein (Zang et al., 2025). However, moisture content had no significant effect on elasticity. When moisture content exceeds 70%, the adhesiveness of HMMA decreases significantly. Higher moisture content is believed to lead to incomplete organization (Lin et al., 2006), which may explain the measurable reduction in texture properties. The increase in moisture content may make the HMMA structure more porous, which potentially result in volatile flavor compounds being more easily dispersed during preparation and storage. This may explain why HMMA-80% exhibits the lowest total volatile compound content in Table 1. Conversely, the moderate hardness of HMMA-60% and HMMA-70% proved more conducive to flavor retention and controlled release, thereby contributing to their harmonious sensory profile.

#### 3.1.3 Effect of moisture content on microstructure of HMMAs

**Fig. 2:**
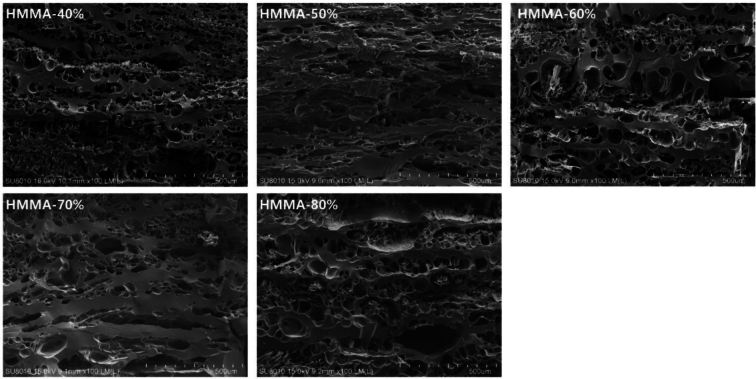
Effects of moisture content on the microstructure of HMMAs.

The microstructure of HMMAs with different moisture content is shown in Fig. 2. The pore structure distribution on the surface of HMMA-40% was extremely uneven, and the layered structure was not prominent. As the moisture content increased, the layered structure of HMMA becomes gradually more pronounced, with the most distinct layered structure observed in HMMA-60% and HMMA-70%. Studies have shown that as moisture content increases, the flowability of raw materials during extrusion was improved, reducing HMMA expansion and increasing structural compactness (Guo et al., 2020). HMMA-80% exhibited a sponge-like structure due to excessive moisture content, lacking a layered structure. HMMA-80% exhibited a spongy structure, which may be due to excessive moisture content and low compression temperature, resulting in insufficient protein denaturation. This caused HMMA to lack a fibrous structure and become more viscous due to the large amount of water filling the protein voids(Kitabatake et al., 2006). This was consistent with the conclusion of 3.1.2. Therefore, the moisture content jointly determines the final texture and flavor expression of the product by regulating the fibrous structure of proteins. Selecting a moisture content of 70% achieves the optimal balance between hardness, fibrous structure, and flavor retention ability.

### 3.2 Effect of extrusion temperature on HMMAs

#### 3.2.1 Effect of extrusion temperature on aroma and sensory profiles of HMMAs

**Fig. 3:**
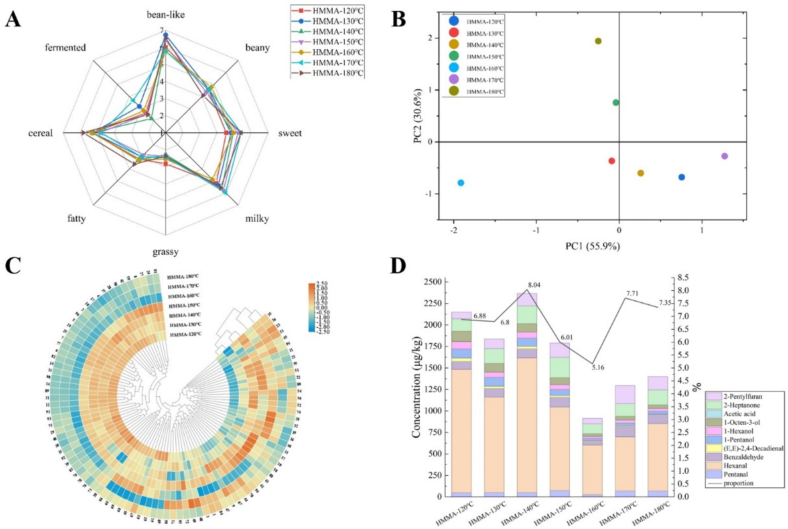
The aroma profiles and compound composition of HMMAs at different extrusion temperatures. (A) The aroma profiles of HMMAs. (B) PCA chart for aroma profiles of HMMAs. (C) Heatmap analysis of aroma compounds in HMMAs. (D) The content and proportion of off-flavor compounds in HMMAs. The numbers correspond to the compound No. in Table 3.

A sensory evaluation analysis was conducted on the overall aroma of HMMA produced at different extrusion temperatures, with the results shown in Fig. 3 A. Extrusion temperature had a significant impact on the bean-like, fermented, cereal, milky, and sweet aroma of HMMA. Both HMMA-170 °C and HMMA-180 °C exhibited the strongest sweet aroma, while HMMA-180 °C also had the strongest cereal and fatty aroma, and the weakest beany aroma. HMMA-170 °C exhibited the strongest milky and fermentation aroma, while having the weakest bean-like and cereal aroma. Both HMMA-160 °C and HMMA-170 °C had the weakest bean-like aroma. The milky aroma of HMMA-160 °C was the weakest, but it exhibited pronounced cereal and fatty aromas with moderate sweet aroma. Beyond the beany aroma, undesirable flavors such as fermentation and grassy aromas are effectively controlled.

To investigate the flavor differences between HMMAs prepared at different extrusion temperatures, HMMAs were subjected to *E*-nose analysis, with the results shown in Fig. 3 B. The total contribution rate of the two principal components (PC1 and PC2) was 86.5%, indicating that the analysis results could reflect the main characteristic information of the samples. The distances in the figure represented the aroma differences between HMMAs, with greater distances indicating larger aroma differences. PC1 clearly distinguished HMMA-160 °C from the other six HMMA samples, indicating that the overall aroma profile of HMMA underwent a significant change at an extrusion temperature of 160 °C.

The volatile compounds of HMMAs prepared with different extrusion temperature were analyzed by SAFE-GC–MS (Table 3). A total of 96 compounds were identified, including 7 aldehydes, 14 alcohols, 8 acids, 11 esters, 10 ketones, 19 hydrocarbons, 22 heterocyclic compounds, 3 phenols, and 2 other compounds. The studies on moisture content and extrusion temperature were conducted in two separate experiments, therefore data for the same sample under identical conditions may exhibit discrepancies. When the extrusion temperature was between 120 °C and 150 °C, the total amount of volatile compounds in HMMAs showed no significant changes. However, when the extrusion temperature reached or exceeded 160 °C, the total amount of compounds decreased significantly. This result is consistent with the findings of (Xun et al., 2019). This may be because, to some extent, increasing the extrusion temperature makes the structure of HMMAs more compact, reducing its susceptibility to moisture evaporation. However, as the extrusion temperature continues to rise, it increases the moisture evaporation rate of HMMAs, thereby releasing more volatile flavor compounds. The content of aldehydes was highest at 140 °C, with hexanal also having the highest content, indicating that lipid oxidation was most active at moderate high temperatures. Beyond 140 °C, the content of aldehydes decreased, possibly due to the inherent instability of aldehydes, which undergo further reactions or decomposition. Total ketone content peaked at 150 °C. Key ketones such as hydroxyacetone and acetoin exhibited a significant increase at this temperature, indicating their role as signature products of high-temperature Maillard reactions. Heterocyclic compounds constituted one of the categories most significantly affected by temperature, maintaining high levels between 120 °C and 150 °C before reaching a maximum at 150 °C. Among them, *N*-methyl-2-pyrrolidone and maltol had extremely high content at 150 °C. However, after exceeding 150 °C, the content of many heterocyclic compounds sharply decreased, such as 2-furanmethanol, indicating that excessively high temperatures exert a profoundly destructive effect on Maillard reaction products.

**Table 3:** Changes in the content of volatile compounds in HMMAs during the extrusion process at different extrusion temperature.

Type	No.	CAS	Compounds	RI	Concentration (μg/kg)							Identification
					HMMA-120 °C	HMMA-130 °C	HMMA-140 °C	HMMA-150 °C	HMMA-160 °C	HMMA-170 °C	HMMA-180 °C	
Aldehydes	1	110–62-3	Pentanal	/	47.99 ± 5.86 ^b^	51.05 ± 4.87 ^b^	50.58 ± 1.88 ^b^	73.91 ± 13.73 ^a^	26.09 ± 2.02 ^c^	68.61 ± 4.31 ^a^	68.91 ± 7.02 ^a^	MS
	2	66–25-1	Hexanal	1077	1434.73 ± 150.72 ^b^	1111.47 ± 58.7 ^c^	1566.78 ± 82.75 ^a^	972.09 ± 37.79 ^d^	577.78 ± 45.28 ^f^	629.67 ± 14.43 ^f^	785.31 ± 36.75 ^e^	MS、RI、S
	3	100–52-7	Benzaldehyde	1515	92.78 ± 4.28 ^d^	99.46 ± 2.39 ^cd^	105.19 ± 6.12 ^c^	113.01 ± 3.98 ^b^	42.68 ± 2.68 ^e^	121.27 ± 4.93 ^a^	105.23 ± 2.63 ^c^	MS、RI、S
	4	25,152–84-5	(E,E)-2,4-Decadienal	1806	40.22 ± 2.39 ^a^	25.76 ± 3.17 ^c^	30.92 ± 1.92 ^b^	20.87 ± 2.15 ^d^	8.89 ± 0.96 ^e^	9.35 ± 0.09 ^e^	4.19 ± 0.51 ^f^	MS、RI、S
	5	121–33-5	Vanillin	2554	546.11 ± 34.25 ^a^	509.19 ± 23.48 ^a^	545.2 ± 40.5 ^a^	493.65 ± 52.78 ^a^	209.1 ± 20.49 ^c^	256.62 ± 12.27 ^bc^	281.08 ± 14.47 ^b^	MS、RI、S
	6	134–96-3	4-Hydroxy-3,5-dimethoxybenzaldehyde	/	184.53 ± 16.68 ^a^	165.51 ± 17.56 ^a^	181.26 ± 14.16 ^a^	184.99 ± 18.09 ^a^	65.93 ± 11.12 ^c^	96.85 ± 9.23 ^b^	101.09 ± 5.22 ^b^	MS
	7	123–08-0	4-Hydroxybenzaldehyde	/	198.6 ± 16.25 ^a^	177.69 ± 10.31 ^a^	191.22 ± 8.88 ^a^	184.18 ± 22.99 ^a^	88.13 ± 17.34 ^b^	88.44 ± 3.36 ^b^	110.54 ± 14.12 ^b^	MS
Subtotal					2544.96 ± 230.43	2140.13 ± 120.48	2671.16 ± 156.21	2042.7 ± 151.51	1018.6 ± 99.89	1270.8 ± 48.62	1456.35 ± 80.72	
Alcohols	8	75–85-4	Amylene hydrate	1015	373.79 ± 5.79 ^b^	327.68 ± 49.56 ^bc^	327 ± 39.82 ^bc^	423.42 ± 18 ^a^	228.97 ± 14.63 ^d^	302.41 ± 9.56 ^c^	306.29 ± 28.37 ^c^	MS、RI、S
	9	71–36-3	1-Butanol	1144	4.77 ± 0.19 ^b^	4.84 ± 0.18 ^b^	6.63 ± 0.27 ^a^	4.84 ± 0.65 ^b^	4.68 ± 0.09 ^b^	6.75 ± 0.23 ^a^	5.3 ± 0.52 ^b^	MS、RI、S
	10	1569-50-2	3-Penten-2-ol	1169	5.3 ± 0.62 ^b^	5.84 ± 0.79 ^b^	6.66 ± 0.67 ^b^	10.64 ± 2.06 ^a^	6.83 ± 0.71 ^b^	7.33 ± 1.18 ^b^	6.01 ± 0.83 ^b^	MS、RI、S
	11	623–37-0	3-Hexanol	1194	9.23 ± 0.86 ^c^	14.28 ± 1.13 ^b^	20.05 ± 2.53 ^a^	20.14 ± 3 ^a^	13.12 ± 2.24 ^b^	11.55 ± 0.56 ^bc^	11.93 ± 2.33 ^bc^	MS、RI、S
	12	71–41-0	1-Pentanol	1247	105.21 ± 7.96 ^a^	103.26 ± 3.11 ^a^	94.06 ± 14.62 ^a^	69.67 ± 1.03 ^b^	24.44 ± 1.26 ^c^	31.42 ± 1.09 ^c^	33.82 ± 1.84 ^c^	MS、RI、S
	13	111–27-3	1-Hexanol	1348	85.89 ± 9.33 ^a^	60.7 ± 2.38 ^c^	70.84 ± 5.58 ^b^	57.28 ± 7.31 ^c^	22.7 ± 0.22 ^e^	32.15 ± 2.36 ^d^	32.31 ± 1.77 ^d^	MS、RI、S
	14	3391-86-4	1-Octen-3-ol	1444	118.96 ± 11.98 ^a^	101.4 ± 8.02 ^bc^	96.95 ± 6.49 ^b^	79.03 ± 2.93 ^c^	31.57 ± 3.03 ^e^	44.51 ± 0.99 ^d^	40.65 ± 1.34 ^de^	MS、RI、S
	15	104–76-7	2-Ethylhexanol	1481	38.6 ± 1.08 ^a^	37.85 ± 2.98 ^a^	28.71 ± 2.17 ^c^	33.73 ± 0.64 ^b^	17.45 ± 1.02 ^e^	25 ± 1.2 ^d^	22.75 ± 1.85 ^d^	MS、RI、S
	16	513–85-9	2,3-Butanediol	1574	13.23 ± 0.31 ^cd^	11.4 ± 1.11 ^d^	15.99 ± 1.68 ^ab^	16.65 ± 2.42 ^ab^	18.08 ± 1.22 ^a^	14.59 ± 0.61 ^bc^	11.67 ± 0.58 ^d^	MS、RI、S
	17	112–34-5	Diethylene glycol monobutyl ether	1778	24.24 ± 2.88 ^b^	29.08 ± 2.06 ^a^	29.18 ± 2.87 ^a^	13.12 ± 0.59 ^c^	5.23 ± 0.55 ^d^	10.21 ± 0.6 ^c^	5.91 ± 0.98 ^d^	MS、RI、S
	18	100–51-6	Benzyl alcohol	1859	451.16 ± 39.45 ^b^	282.1 ± 13.97 ^c^	676.02 ± 56.41 ^a^	239.97 ± 8.9 ^c^	99.43 ± 3.91 ^d^	127.45 ± 4.22 ^d^	86.7 ± 3.13 ^d^	MS、RI、S
	19	60–12-8	Phenylethyl alcohol	1891	32.65 ± 2.73 ^e^	42.01 ± 2.67 ^d^	56.85 ± 11.12 ^c^	71.75 ± 10.22 ^b^	106.43 ± 6.76 ^a^	47.2 ± 5.1 ^d^	26.8 ± 1.62 ^e^	MS、RI、S
	20	122–99-6	2-Phenoxyethanol	2124	99.98 ± 6.54 ^a^	68.98 ± 3.89 ^c^	88.6 ± 7.92 ^b^	104.3 ± 2.57 ^a^	45.19 ± 3.46 ^d^	41.06 ± 0.7 ^d^	41.25 ± 0.9 ^d^	MS、RI、S
	21	36,653–82-4	1-Hexadecanol	2363	2.56 ± 0.12 ^d^	3.02 ± 0.25 ^bcd^	3.65 ± 0.33 ^ab^	3.71 ± 0.29 ^a^	3.36 ± 0.65 ^abc^	3.02 ± 0.33 ^bcd^	2.78 ± 0.08 ^cd^	MS、RI、S
Subtotal					1365.57 ± 89.83	1092.43 ± 92.1	1521.19 ± 152.48	1148.26 ± 60.61	627.48 ± 39.76	704.65 ± 28.73	634.17 ± 46.14	
Acids	22	64–19-7	Acetic acid	1452	0.62 ± 0.1 ^b^	0.74 ± 0.12 ^ab^	0.78 ± 0.11 ^ab^	0.85 ± 0.16 ^a^	0.72 ± 0.12 ^ab^	0.77 ± 0.06 ^ab^	0.68 ± 0.1 ^ab^	MS、RI、S
	23	109–52-4	Pentanoic acid	1731	0.47 ± 0.03 ^c^	0.54 ± 0.06 ^bc^	0.45 ± 0.03 ^c^	0.64 ± 0.02 ^ab^	0.53 ± 0.06 ^bc^	0.67 ± 0.12 ^a^	0.74 ± 0.06 ^a^	MS、RI、S
	24	142–62-1	Hexanoic acid	1833	291.87 ± 3.69 ^a^	273.52 ± 26.13 ^ab^	282.93 ± 40.83 ^ab^	251.29 ± 13.36 ^b^	110.74 ± 8.76 ^c^	126.48 ± 3.12 ^c^	140.26 ± 14.06 ^c^	MS、RI、S
	25	124–07-2	Octanoic acid	2046	80.48 ± 3.09 ^a^	80.91 ± 3.34 ^a^	79.12 ± 8.78 ^ab^	72.8 ± 2.5 ^b^	34.54 ± 2.33 ^d^	43.21 ± 1 ^c^	49.78 ± 1.93 ^c^	MS、RI、S
	26	112–05-0	Nonanoic acid	2153	28.79 ± 5.26 ^bcd^	32.96 ± 4.28 ^bc^	36.82 ± 4.81 ^ab^	42.26 ± 7.76 ^a^	21.29 ± 2.94 ^d^	23.94 ± 3.51 ^d^	25.31 ± 1.01 ^cd^	MS、RI、S
	27	1871-67-6	(E)-2-Octenoicacid	2173	17.2 ± 0.15 ^bc^	18.99 ± 1.05 ^bc^	19.33 ± 1.62 ^b^	25.33 ± 0.9 ^a^	12.15 ± 1.84 ^d^	16.12 ± 0.86 ^c^	16.96 ± 1.61 ^bc^	MS、RI、S
	28	334–48-5	Decanoic acid	2258	4.84 ± 0.64 ^e^	6.22 ± 0.36 ^d^	8.1 ± 1.09 ^bc^	7.04 ± 0.41 ^cd^	15.32 ± 0.52 ^a^	8.91 ± 0.28 ^b^	6.63 ± 0.88 ^d^	MS、RI、S
	29	57–10-3	Hexadecanoic acid	/	1183.72 ± 195.38 ^a^	892.48 ± 54.42 ^bc^	1118.62 ± 64.88 ^a^	1061.75 ± 166 ^ab^	245.51 ± 11.8 ^d^	513.69 ± 55.18 ^c^	581.48 ± 87.42 ^c^	MS
Subtotal					1607.99 ± 208.36	1306.36 ± 89.76	1546.15 ± 122.15	1461.97 ± 191.12	440.8 ± 28.37	733.8 ± 64.12	821.83 ± 107.08	
Esters	30	96–48-0	γ-Butyrolactone	1614	139.26 ± 26.38 ^a^	94.18 ± 3.42 ^bc^	10.34 ± 0.9 ^d^	55.24 ± 0.79 ^c^	40.86 ± 2.33 ^c^	57.17 ± 1.36 ^c^	39.85 ± 1.26 ^c^	MS、RI、S
	31	695–06-7	5-Ethyldihydro-2(3H)-furanone	1686	22.64 ± 2.56 ^a^	21.03 ± 1.83 ^a^	21.09 ± 1.54 ^a^	15.65 ± 0.79 ^b^	7.13 ± 0.37 ^c^	8.43 ± 0.56 ^c^	8.34 ± 0.23 ^c^	MS、RI、S
	32	923–26-2	2-Hydroxypropyl methacrylate	1724	0.62 ± 0.02 ^f^	0.82 ± 0.05 ^e^	1.34 ± 0.07 ^b^	0.97 ± 0.09 ^cd^	4.17 ± 0.09 ^a^	1.04 ± 0.09 ^c^	0.89 ± 0.05 ^de^	MS、RI、S
	33	104–50-7	γ-Octanoic lactone	1894	0.29 ± 0.02 ^d^	0.9 ± 0.09 ^b^	0.75 ± 0.06 ^bc^	1.89 ± 0.33 ^a^	0.55 ± 0.01 ^c^	0.2 ± 0.04 ^d^	0.28 ± 0.03 ^d^	MS、RI
	34	104–61-0	γ-Nonanolactone	2005	74.94 ± 4.81 ^a^	57.43 ± 3.87 ^c^	63.24 ± 3.57 ^b^	46.43 ± 0.66 ^d^	20.83 ± 1.07 ^e^	22.57 ± 0.93 ^e^	24.36 ± 1.01 ^e^	MS、RI、S
	35	112–39-0	Hexadecanoic acid, methyl ester	2195	9.18 ± 0.85 ^c^	4.04 ± 0.51 ^d^	12.81 ± 1.76 ^b^	15.55 ± 2.39 ^a^	9.53 ± 1.21 ^c^	12.58 ± 0.78 ^b^	12.71 ± 0.9 ^b^	MS、RI、S
	36	628–97-7	Hexadecanoic acid, ethyl ester	2236	1.48 ± 0.29 ^d^	0.89 ± 0.06 ^e^	1.85 ± 0.27 ^cd^	3.04 ± 0.6 ^a^	1.89 ± 0.31 ^bcd^	2.47 ± 0.21 ^b^	2.35 ± 0.23 ^bc^	MS、RI、S
	37	131–11–3	Dimethyl phthalate	2277	64.34 ± 2.94 ^a^	50.27 ± 2.07 ^bc^	61.29 ± 5.69 ^a^	41.04 ± 0.88 ^c^	20.64 ± 1.56 ^e^	26.7 ± 1.1 ^d^	19.05 ± 0.72 ^e^	MS、RI、S
	38	84–69-5	Diisobutyl phthalate	2520	163.22 ± 26.61 ^a^	104.55 ± 15.7 ^bc^	72.97 ± 13.25 ^c^	83.64 ± 10.48 ^bc^	27.22 ± 1.96 ^d^	40.19 ± 6.76 ^d^	27.65 ± 5.41 ^d^	MS、RI、S
	39	5469-16-9	(+/−)-3-hydroxy-γ-butyrolactone	2592	324.87 ± 12.11 ^a^	242.72 ± 5.31 ^c^	233.02 ± 25.55 ^c^	269.14 ± 15.7 ^b^	91.65 ± 1.25 ^e^	126.68 ± 5.57 ^d^	136.27 ± 7.7 ^d^	MS、RI、S
	40	84–74-2	Dibutyl phthalate	2676	960.22 ± 70.47 ^a^	572.93 ± 56.49 ^bc^	460.6 ± 75.1 ^c^	513.72 ± 50.12 ^bc^	194.56 ± 17.96 ^e^	304.5 ± 40.03 ^d^	250.5 ± 25.39 ^de^	MS、RI、S
Subtotal					1761.06 ± 147.06	1149.75 ± 89.4	939.31 ± 127.76	1046.31 ± 82.83	419.04 ± 28.13	602.53 ± 57.43	522.25 ± 42.93	
Ketones	41	110–43-0	2-Heptanone	1179	145.91 ± 13.22 ^d^	171.7 ± 9.83 ^c^	206.79 ± 14.92 ^b^	237.24 ± 5.38 ^a^	113.91 ± 13.92 ^e^	147.3 ± 4.05 ^d^	173.37 ± 6.78 ^c^	MS、RI、S
	42	513–86-0	Acetoin	1275	184.46 ± 11.93 ^b^	159.72 ± 4.76 ^c^	174.66 ± 13.89 ^bc^	210.14 ± 28.14 ^a^	87.03 ± 0.22 ^e^	112.17 ± 4.92 ^d^	116.28 ± 4.41 ^d^	MS、RI、S
	43	116–09-6	Hydroxyacetone	1289	329.86 ± 39.14 ^c^	304.85 ± 23.94 ^c^	410.07 ± 29.84 ^b^	562.47 ± 8.15 ^a^	240.16 ± 6.82 ^d^	336 ± 2.28 ^c^	341.03 ± 4.17 ^c^	MS、RI、S
	44	1120-73-6	2-methylcyclopentenone	1359	1.39 ± 0.11 ^e^	1.97 ± 0.09 ^d^	2.95 ± 0.48 ^a^	2.69 ± 0.02 ^ab^	1.18 ± 0.11 ^e^	2.41 ± 0.06 ^bc^	2.09 ± 0.1 ^cd^	MS、RI、S
	45	5077-67-8	1-Hydroxy-2-butanone	1366	3.39 ± 0.2 ^d^	5.14 ± 0.93 ^c^	8.15 ± 1.15 ^a^	6.65 ± 0.55 ^b^	5.85 ± 0.88 ^bc^	6.59 ± 0.27 ^b^	5.58 ± 0.55 ^bc^	MS、RI、S
	46	30,086–02-3	3,5-Octadien-2-one	1561	23.24 ± 0.61 ^c^	22.03 ± 0.53 ^c^	27.24 ± 0.84 ^b^	28.57 ± 4.41 ^b^	32.66 ± 1.4 ^a^	27.47 ± 1.2 ^b^	21.31 ± 0.77 ^c^	MS、RI、S
	47	98–86-2	Acetophenone	1632	2.1 ± 0.11 ^c^	2.04 ± 0.2 ^c^	3.7 ± 0.13 ^a^	2.46 ± 0.14 ^b^	1.17 ± 0.09 ^d^	2.43 ± 0.07 ^b^	2.24 ± 0.24 ^bc^	MS、RI、S
	48	80–71-7	Methyl cyclopentenolone	1816	5.05 ± 0.71 ^bc^	3.74 ± 0.31 ^d^	4.4 ± 0.53 ^cd^	5.8 ± 0.38 ^b^	4.73 ± 0.52 ^cd^	7.48 ± 0.63 ^a^	4.24 ± 0.75 ^cd^	MS、RI、S
	49	21,835–01-8	3-Ethyl-2-hydroxy-2-cyclopenten-1-one	1879	9.26 ± 0.54 ^c^	9.64 ± 1.24 ^c^	11.08 ± 1.15 ^bc^	12.85 ± 1.32 ^b^	10.63 ± 0.41 ^c^	24.19 ± 1.97 ^a^	10.08 ± 0.57 ^c^	MS、RI、S
	50	498–02-2	Apocynin	2626	21.95 ± 3.34 ^a^	17.8 ± 1.41 ^bc^	18.95 ± 0.57 ^ab^	20.19 ± 0.4 ^ab^	9.8 ± 1.67 ^c^	10.69 ± 1.69 ^c^	10.42 ± 1.29 ^c^	MS、RI、S
Subtotal					726.61 ± 69.9	698.64 ± 43.23	867.99 ± 63.49	1089.07 ± 48.89	507.12 ± 26.05	676.72 ± 17.14	686.64 ± 19.61	
Hydrocarbons	51	15,869–93-9	3,5-Dimethyloctane	1042	0.89 ± 0.07 ^e^	2 ± 0.29 ^d^	3.48 ± 0.28 ^b^	8.03 ± 0.3 ^a^	3.82 ± 0.3 ^b^	2.65 ± 0.34 ^c^	3.68 ± 0.48 ^b^	MS
	52	6975-98-0	2-Methyldecane	1061	2.12 ± 0.35 ^e^	4.94 ± 0.53 ^c^	10.06 ± 0.78 ^a^	4.49 ± 0.43 ^cd^	7.02 ± 1.04 ^b^	3.26 ± 0.46 ^d^	6.11 ± 0.1 ^b^	MS
	53	1120-21-4	Undecane	1100	–	–	–	–	71.79 ± 10.2 ^c^	85.92 ± 3.04 ^b^	98.66 ± 1.58 ^a^	MS、RI、S
	54	100–41-4	Ethylbenzene	1119	215.01 ± 6.5 ^e^	377.61 ± 60.41 ^d^	726.89 ± 129 ^a^	566.49 ± 98.05 ^bc^	635.04 ± 59.03 ^ab^	201.83 ± 2.59 ^e^	473 ± 91.38 ^cd^	MS、RI、S
	55	112–40-3	Dodecane	1199	2.87 ± 0.31 ^d^	5.13 ± 0.69 ^bc^	7.81 ± 1.04 ^a^	8.71 ± 1.72 ^a^	5.99 ± 0.06 ^b^	3.75 ± 0.5 ^cd^	3.83 ± 0.63 ^cd^	MS、RI、S
	56	100–42-5	Styrene	1247	56.44 ± 1.52 ^e^	116.39 ± 20.19 ^cd^	234.21 ± 46.37 ^a^	93.06 ± 8.99 ^de^	189.33 ± 15.03 ^b^	57.27 ± 0.52 ^e^	134.97 ± 23.89 ^c^	MS、RI、S
	57	544–76-3	Hexadecane	1594	4.3 ± 0.78 ^c^	3.01 ± 0.39 ^c^	2.95 ± 0.45 ^c^	24.95 ± 2.56 ^a^	6.16 ± 0.54 ^b^	4.26 ± 0.13 ^c^	3.4 ± 0.39 ^c^	MS、RI、S
	58	629–78-7	Heptadecane	1697	1.65 ± 0.05 ^c^	2.74 ± 0.49 ^bc^	3.07 ± 0.49 ^b^	9.94 ± 1.8 ^a^	3.36 ± 0.64 ^b^	2.8 ± 0.41 ^bc^	2.96 ± 0.55 ^b^	MS、RI、S
	59	593–45-3	Octacosane	1793	14.76 ± 2.8 ^b^	13.3 ± 0.3 ^bc^	15.65 ± 1.81 ^ab^	18.46 ± 2.57 ^a^	10.22 ± 1.22 ^cd^	9.86 ± 0.62 ^d^	11.26 ± 0.53 ^cd^	MS、RI、S
	60	629–92-5	Nonadecane	1889	101.45 ± 11.75 ^a^	71.19 ± 2.64 ^bc^	77.25 ± 13.35 ^b^	70.78 ± 8.97 ^bc^	53.31 ± 10.07 ^de^	41.06 ± 2.13 ^e^	60.36 ± 1.13 ^cd^	MS、RI、S
	61	112–95-8	Eicosane	1989	413.12 ± 45.77 ^a^	268.67 ± 25.94 ^b^	337.64 ± 45.02 ^b^	293.07 ± 56.8 ^b^	320.24 ± 47.71 ^b^	171.61 ± 21.28 ^c^	260.79 ± 23.07 ^b^	MS、RI、S
	62	629–94-7	Heneicosane	2090	557.9 ± 69.81 ^a^	389.78 ± 45.24 ^b^	414.44 ± 71.1 ^b^	427.88 ± 82.14 ^b^	393.08 ± 72.47 ^b^	247.63 ± 42 ^c^	396.77 ± 50.56 ^b^	MS、RI、S
	63	629–97-0	Docosane	2189	548.07 ± 93.73 ^a^	417.75 ± 58.8 ^b^	452.07 ± 36.54 ^ab^	462.81 ± 88.16 ^ab^	441.17 ± 38.07 ^ab^	249.44 ± 35.35 ^c^	427.31 ± 54.17 ^b^	MS、RI、S
	64	638–67-5	Tricosane	2289	617.88 ± 116.61 ^ab^	473.41 ± 80.93 ^bc^	506.47 ± 18.69 ^bc^	454.09 ± 77.56 ^c^	720.16 ± 122.31 ^a^	216.32 ± 35 ^d^	421.38 ± 65.5 ^c^	MS、RI、S
	65	646–31-1	Tetracosane	2389	586.08 ± 17.53 ^ab^	380.27 ± 68.37 ^c^	466.25 ± 33.07 ^bc^	498.98 ± 96.66 ^bc^	720.5 ± 142.29 ^a^	219.37 ± 42.29 ^d^	480.44 ± 95.03 ^bc^	MS、RI、S
	66	629–99-2	Pentacosane	2487	767.36 ± 33.76 ^a^	527.06 ± 103.52 ^b^	566.46 ± 49.65 ^b^	497.55 ± 86.88 ^b^	826.39 ± 145.21 ^a^	229.59 ± 18.32 ^c^	545.93 ± 94.65 ^b^	MS、RI、S
	67	630–01–3	Hexacosane	2585	606.33 ± 72.55 ^bc^	448.09 ± 17.45 ^d^	484.7 ± 72.54 ^cd^	512.68 ± 101.03 ^cd^	791.09 ± 120.09 ^a^	392.02 ± 47.62 ^d^	689.51 ± 96.22 ^ab^	MS、RI、S
	68	593–49-7	Heptacosane	2685	718.82 ± 69.26 ^ab^	522.45 ± 41.63 ^c^	524.87 ± 77.63 ^c^	592.46 ± 116.61 ^bc^	798.39 ± 97.55 ^a^	207.47 ± 33.56 ^d^	501.11 ± 70.18 ^c^	MS、RI、S
	69	630–02-4	Octacosane	2785	581.82 ± 94.38 ^ab^	431.61 ± 23.78 ^c^	455.47 ± 82.81 ^bc^	490.61 ± 92.54 ^bc^	665.3 ± 78.06 ^a^	167.22 ± 30.88 ^d^	417.89 ± 53.22 ^c^	MS、RI、S
Subtotal					5796.88 ± 637.54	4455.4 ± 551.58	5289.74 ± 680.62	5035.05 ± 923.77	6662.37 ± 961.87	2513.32 ± 317.05	4939.37 ± 723.26	
heterocycles	70	3777-69-3	2-Pentylfuran	1222	77.14 ± 4.84 ^e^	110.82 ± 3.73 ^d^	144.33 ± 1.49 ^c^	165.47 ± 8.81 ^b^	63.9 ± 2.05 ^e^	209.49 ± 11.64 ^a^	155.16 ± 12.14 ^bc^	MS、RI、S
	71	98–01-1	Furfural	1458	26.69 ± 1.43 ^c^	33.87 ± 2.29 ^b^	15.38 ± 1.67 ^d^	32.73 ± 2.51 ^b^	19.57 ± 3.66 ^d^	42.12 ± 2.46 ^a^	29.9 ± 2.24 ^bc^	MS、RI、S
	72	1192-62-7	2-Acetylfuran	1495	8.3 ± 0.54 ^c^	9.02 ± 0.55 ^c^	3.45 ± 0.27 ^d^	26.6 ± 4.88 ^a^	6.61 ± 0.3 ^cd^	13.05 ± 0.98 ^b^	6.77 ± 1.07 ^cd^	MS、RI、S
	73	98–00-0	2-Furanmethanol	1653	3548.21 ± 262.16 ^a^	2914.04 ± 148.61 ^b^	714.3 ± 57.34 ^f^	2594.58 ± 11.7 ^c^	1663.15 ± 66.79 ^e^	2328.98 ± 66.02 ^d^	1747.9 ± 69.81 ^e^	MS、RI、S
	74	3857-25-8	(5-Methyl-2-furyl)methanol	1715	9.57 ± 0.2 ^f^	23.34 ± 4.53 ^d^	65.6 ± 1.94 ^a^	23.22 ± 2.65 ^d^	36.86 ± 3.97 ^b^	15.45 ± 0.93 ^e^	31.22 ± 2.5 ^c^	MS、RI、S
	75	497–23-4	2(5H)-Furanone	1736	310.03 ± 36.17 ^a^	260.74 ± 15.87 ^b^	270.64 ± 18.67 ^b^	258.39 ± 2.99 ^b^	130.49 ± 6.63 ^d^	192.86 ± 5.4 ^c^	185.45 ± 6.79 ^c^	MS、RI、S
	76	636–72-6	2-Thiophenemethanol	1925	27.82 ± 1.84 ^a^	13.63 ± 1.37 ^b^	9.84 ± 0.05 ^c^	12.04 ± 0.98 ^b^	6.87 ± 0.21 ^d^	8.66 ± 0.43 ^c^	6.72 ± 0.46 ^d^	MS、RI、S
	77	118–71-8	Maltol	1951	10,317.27 ± 540.41 ^b^	10,650.52 ± 553.13 ^b^	11,808.99 ± 1127.56 ^a^	10,658.23 ± 202.27 ^b^	4235.27 ± 244.27 ^d^	5528.86 ± 183.7 ^c^	5791.74 ± 197.66 ^c^	MS、RI、S
	78	17,678–19-2	2-Furylhydroxymethylketone	1981	72.26 ± 2.81 ^a^	60.06 ± 5.39 ^b^	39.73 ± 4.31 ^c^	56.67 ± 2.18 ^b^	28.56 ± 1.51 ^d^	41.69 ± 0.85 ^c^	43.19 ± 1.63 ^c^	MS、RI、S
	79	3658-77-3	Furaneol	2021	39.45 ± 1.04 ^a^	34.61 ± 2.07 ^b^	16.51 ± 1.37 ^e^	28.91 ± 0.84 ^c^	14.15 ± 0.97 ^f^	19.49 ± 0.58 ^d^	16.39 ± 0.61 ^e^	MS、RI、S
	80	19,322–27-1	4-Hydroxy-5-methyl-3-furanone	2105	8.35 ± 0.27 ^d^	10.35 ± 0.59 ^c^	12.46 ± 0.7 ^b^	15.26 ± 0.37 ^a^	11.94 ± 1.01 ^b^	12.8 ± 0.56 ^b^	9.61 ± 0.35 ^c^	MS、RI、S
	81	28,564–83-2	2,3-Dihydro-3,5-dihydroxy-6-methyl-4(H)-pyran-4-one	2249	225.27 ± 14.39 ^c^	244.92 ± 11.98 ^bc^	252.62 ± 22.13 ^b^	305.97 ± 11.15 ^a^	137.46 ± 1.64 ^e^	180.25 ± 8.28 ^d^	187.13 ± 8.63 ^d^	MS、RI、S
	82	496–16-2	2,3-Dihydrobenzofuran	2380	155.1 ± 7.13 ^a^	141.98 ± 6.84 ^b^	133.1 ± 9.69 ^bc^	124 ± 4.93 ^c^	57.93 ± 3.13 ^e^	74.45 ± 2.42 ^d^	79.23 ± 3.18 ^d^	MS
	83	32,780–06-6	(*S*)-5-Hydroxymethyldihydrofuran-2-one	2469	162.87 ± 10.28 ^a^	99.88 ± 7.42 ^b^	93.03 ± 7.09 ^b^	95.71 ± 11.6 ^b^	40.64 ± 0.84 ^d^	58.24 ± 3.58 ^c^	61.6 ± 3.55 ^c^	MS、RI、S
	84	67–47-0	5-Hydroxymethylfurfural	2492	21.5 ± 2.88 ^c^	22.61 ± 1.03 ^bc^	14.31 ± 1.9 ^d^	25.17 ± 1.05 ^abc^	6.43 ± 0.32 ^e^	27.54 ± 4.14 ^a^	25.64 ± 0.89 ^ab^	MS、RI、S
	85	290–37–9	Pyrazine	1206	14.66 ± 1.14 ^c^	18.28 ± 0.88 ^b^	11.79 ± 0.56 ^d^	18.99 ± 0.44 ^b^	8.13 ± 0.75 ^e^	22 ± 0.38 ^a^	12.93 ± 0.78 ^d^	MS、RI、S
	86	109–08-0	2-Methylpyrazine	1259	10.82 ± 0.57 ^c^	11.36 ± 0.45 ^c^	5.3 ± 0.1 ^f^	14.75 ± 0.75 ^b^	8.08 ± 0.82 ^e^	20.31 ± 0.05 ^a^	9.29 ± 0.71 ^d^	MS、RI、S
	87	123–32-0	2,5-Dimethyl pyrazine	1322	4.36 ± 0.2 ^e^	5.71 ± 0.39 ^d^	10.21 ± 1.16 ^a^	7.54 ± 0.53 ^c^	3.67 ± 0.6 ^e^	9.03 ± 0.57 ^b^	6.97 ± 0.58 ^c^	MS、RI、S
	88	872–50-4	N-Methyl-2-pyrrolidone	1668	1799.51 ± 344.5 ^c^	1001.08 ± 68.8 ^d^	2450.39 ± 432.68 ^b^	3020.96 ± 50.26 ^a^	1329.3 ± 78.85 ^d^	1212.58 ± 44.61 ^d^	1316.28 ± 62.44 ^d^	MS、RI、S
	89	616–45-5	2-Pyrrolidinone	2027	75.3 ± 2.17 ^a^	60.16 ± 6.07 ^b^	56.79 ± 5.6 ^b^	50.06 ± 1.18 ^c^	19.85 ± 1.62 ^d^	22.62 ± 0.34 ^d^	23.88 ± 1.43 ^d^	MS、RI、S
	90	120–72-9	Indole	2417	167.11 ± 10.42 ^a^	127.51 ± 6.2 ^c^	146.86 ± 12.87 ^b^	97.44 ± 3.93 ^d^	38.1 ± 2.11 ^e^	42.97 ± 1.24 ^e^	43.22 ± 1.29 ^e^	MS、RI、S
	91	59–48-3	Oxindole	/	164.94 ± 8.95 ^a^	130.61 ± 7.58 ^b^	125.07 ± 12.86 ^b^	126.85 ± 19.86 ^b^	40.9 ± 4.13 ^d^	74.1 ± 7.11 ^c^	72.18 ± 3.75 ^c^	MS、RI、S
Subtotal					17,246.53 ± 1254.34	15,985.1 ± 855.77	16,400.7 ± 1722	17,759.55 ± 345.85	7907.86 ± 426.18	10,157.54 ± 346.26	9862.39 ± 382.48	
Phenols	92	106–44-5	p-Cresol	2068	19.28 ± 3.3 ^a^	16.81 ± 0.52 ^ab^	15.32 ± 1.54 ^b^	15.62 ± 1.24 ^b^	8.71 ± 1.27 ^d^	10.69 ± 0.34 ^cd^	11.91 ± 0.37 ^c^	MS、RI、S
	93	7786-61-0	2-Methoxy-4-vinylphenol	2179	102.53 ± 9.49 ^a^	92.98 ± 5.85 ^a^	98.4 ± 8.34 ^a^	77.19 ± 1.92 ^b^	35.71 ± 2.39 ^c^	41.93 ± 1.98 ^c^	45.57 ± 2.21 ^c^	MS、RI、S
	94	96–76-4	2,4-Di-tert-butylphenol	2297	37.13 ± 1.49 ^de^	40.26 ± 2.42 ^cde^	49.25 ± 1.13 ^ab^	51.27 ± 3.73 ^a^	34.14 ± 4.66 ^e^	41.43 ± 5.06 ^cd^	44.1 ± 3.16 ^bc^	MS、RI、S
Subtotal					158.94 ± 14.28	150.05 ± 8.79	162.97 ± 11.01	144.08 ± 6.89	78.56 ± 8.32	94.05 ± 7.38	101.58 ± 5.74	
Others	95	60–35-5	Acetamide	1771	5.88 ± 0.38 ^c^	7.88 ± 1.52 ^b^	8.55 ± 0.55 ^ab^	9.36 ± 0.94 ^ab^	9.37 ± 0.33 ^ab^	10.2 ± 0.52 ^a^	8.27 ± 1.41 ^b^	MS、RI、S
	96	541–46-8	Isovaleramide	1912	34.21 ± 2.16 ^a^	34.34 ± 0.95 ^a^	35.64 ± 1.48 ^a^	31.83 ± 0.78 ^b^	13.74 ± 0.88 ^d^	16.71 ± 1.57 ^c^	17.57 ± 0.55 ^c^	MS、RI、S
Subtotal					40.09 ± 2.54	42.22 ± 2.47	44.19 ± 2.03	41.19 ± 1.72	23.11 ± 1.21	26.91 ± 2.09	25.84 ± 1.96	
Total					31,248.63 ± 2654.28	27,020.08 ± 1853.58	29,443.4 ± 3037.76	29,768.18 ± 1813.19	17,684.94 ± 1619.77	16,780.32 ± 888.82	19,050.41 ± 1409.91	

-, Not detected. Note: Different letters in each row indicate significance (*p* < 0.05).

HCA analysis (Fig. 3 C) showed that HMMA-120 °C, HMMA-130 °C, HMMA-140 °C, and HMMA-150 °C clustered into one class, while HMMA-160 °C, HMMA-170 °C, and HMMA-180 °C clustered into another. This indicates that the effect of extrusion temperature on HMMA is non-linear, with 160 °C representing a critical temperature inflection point during extrusion. HCA analysis distinguished HMMA-120 °C from HMMA-170 °C, yet *E*-nose results placed both samples in the same quadrant, indicating that they had similar aroma characteristics. This is because HCA categorises samples based on the overall similarity of concentrations across all detected volatile compounds, indicating a clear distinction in chemical composition at the weight level between high-temperature and low-temperature samples. In contrast, E-nose PCA analysis detected the response signals of the overall aroma profiles of the samples. Sensors exhibit broad-spectrum yet selectively focused sensitivity to different types of volatile compounds, making their results more reflective of the similarity in the overall aroma characteristics of the samples. Table 3 also indicated that the proportion of each compound relative to the total volatile compounds was similar in both HMMA-120 °C and HMMA-170 °C samples. This similarity in compound proportions may also contribute to the similarity in aroma profiles between these two samples. Fig. 3 D showed that the content of off-flavor compounds also decreased significantly when the extrusion temperature exceeded 160 °C. The proportion of off-flavor compounds in HMMA-160 °C was the smallest (5.16%) among all volatile compounds. The above results indicates that 160 °C is a turning point in the extrusion process, consistent with the results in Fig. 3 B.

#### 3.2.2 Effect of extrusion temperature on texture analysis of HMMAs

Increasing the extrusion temperature facilitates the transformation of proteins into a molten state during the extrusion process, enabling thorough shear mixing of the raw materials within the barrel (Han, 2023). At higher temperatures, interactions between proteins and between proteins and water are enhanced, as elevated temperatures accelerate protein polymerization reactions, thereby promoting the formation of disulfide bonds. However, excessive heating may also impair product hardness, chewiness, and elasticity, affecting product quality and flavor (Chao et al., 2025). Texture analysis of HMMA at different extrusion temperatures were shown in Table S2. HMMA-140 °C exhibited the highest hardness and adhesiveness, along with strong chewiness. Protein interactions and organization within the sample influence its hardness, while chewiness represents the energy required to chew the sample and facilitate swallowing (Pu et al., 2024). This indicates that protein interactions in HMMAs are strongest at an extrusion temperature of 140 °C. HMMA-180 °C exhibited the lowest hardness, adhesiveness, chewability, and adhesion, indicating poor texture when the extrusion temperature reached 180 °C. However, extrusion temperature had no significant effect on the elasticity of HMMAs, consistent with the findings of (Grahl et al., 2018). It has been shown that increasing the extruder barrel temperature from 110 °C to 140 °C has no effect on the texture of TVP. Combined with Table 3, it can be observed that when the extrusion temperature reaches 160 °C, the samples' hardness and chewiness remain significantly higher than those processed at 120–130 °C. This maintained sufficient textural strength while avoiding the formation of off-flavor compounds that may arise from excessive heating, thereby achieving a balance between texture and flavor.

#### 3.2.3 Effect of extrusion temperature on microstructure of HMMAs

**Fig. 4:**
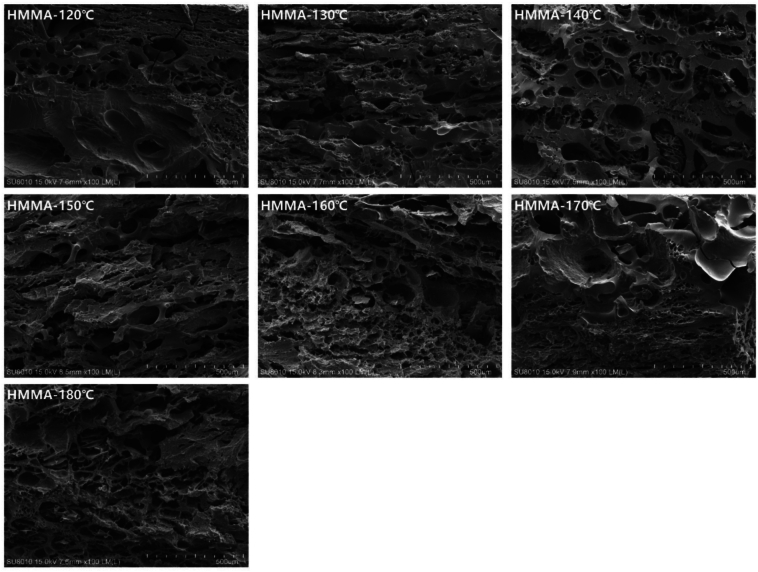
Effects of extrusion temperature on the microstructure of HMMAs.

Temperature is a key factor in the extrusion process, as it causes protein structural deformation, promotes the aggregation of functional proteins and disulfide bond cross-linking, and ensures water evaporation to form the final texture. Research has shown that at a moisture content of 70%, as temperature increased, the structure became more organized, which may be due to increased extrusion temperature, leading to higher product temperature and greater protein texturization (Lin et al., 2006). However, excessively high temperatures can also lead to protein degradation and the breakdown of intermolecular bonds, thereby hindering the formation of fibrous structures (Sui et al., 2024). As shown in Fig. 4, at extrusion temperatures between 120 and 140 °C, lower temperatures resulted in HMMAs lacking distinct fiber structures. As the extrusion temperature increased, HMMAs exhibited a distinct layered structure at 150–170 °C. This is because, under appropriate high-temperature conditions, proteins and water fully combine to form a stronger protein network structure. As the temperature continued to rise, excessively high temperatures could disrupt protein structure, causing the HMMAs fibers to become loose (Han, 2023). This was consistent with the findings in 3.2.2 that HMMA exhibited the lowest hardness, adhesiveness, and chewiness at 180 °C.

### 3.3 Correlation between volatile compounds and sensory attributes of HMMA by different extrusion processes

**Fig. 5:**
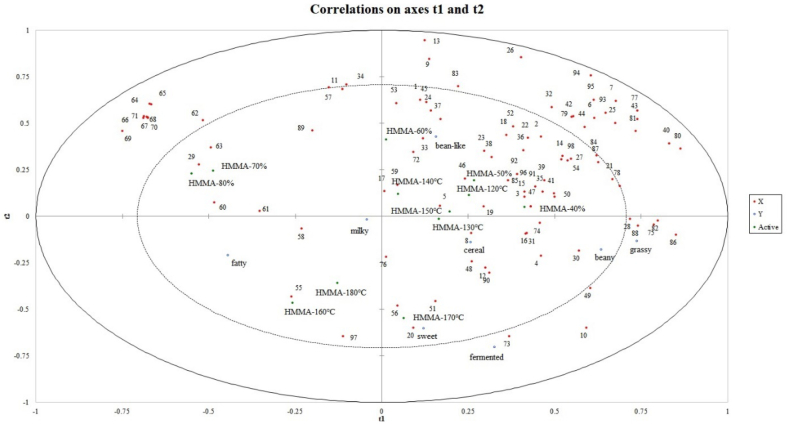
PLSR correlation loading plot of the relationships between 98 volatile compounds (red plots) and sensory attributes. (For interpretation of the references to color in this figure legend, the reader is referred to the web version of this article.)

To more clearly compare the aroma characteristics of HMMAs prepared using different extrusion processes, PLSR analysis was performed on 98 volatile compounds and sensory attributes. As shown in Fig. 5, the X variable (relative volatile compound content) and Y variable (intensity of sensory attributes) were located within the circle. Sensory attributes and volatile compounds that are closely adjacent to each other indicate a strong correlation between the two (Liu et al., 2022). Bean-like aroma is located in the upper-right corner of the load diagram, showing a correlation with HMMA-40%, HMMA-50%, HMMA-60%, HMMA-120 °C, HMMA-140 °C, and HMMA-150 °C, and a significant correlation with odor compounds such as pentanal, hexanal, (E,E)-2,4-decadienal, pentanol, hexanol, 1-octen-3-ol, acetic acid, 2-heptanone, and 2-pentylfuran. Beany, grassy, cereal, fermented and sweet aromas are located in the lower-right corner of the load diagram, showing a strong correlation with HMMA-130 °C and HMMA-170 °C, as well as with odor compounds such as benzaldehyde. Fatty and milky aromas are located in the lower-left region. These two descriptors are positive descriptions in HMMAs, showing a correlation with HMMA-160 °C, and HMMA-180 °C, and no correlation with off-flavor compounds. HMMA-70% and HMMA-80% are situated in the upper-left region and are not associated with flavor or odor compounds. PLSR results also indicate that HMMAs prepared at a moisture content of 70% and an extrusion temperature of 160 °C has a low correlation with off-flavor compounds and is the optimal preparation process.

### 3.4 Identification of volatile compounds in SPI

To determine the influence of materials on HMMA aroma, SPI underwent GC–MS analysis. A total of 49 compounds were identified in SPI (Table S3), including 2 aldehydes, 5 alcohols, 9 acids, 5 esters, 2 heterocyclic compounds, 1 phenol, and 25 hydrocarbons. Compared with SPI, 98 compounds were identified in HMMA, exhibiting significantly greater diversity and complexity than SPI. Moreover, 63 compounds were detected in HMMA but not in SPI, demonstrating that these compounds were newly formed under the high-temperature, high-shear conditions of the extrusion process. Only 35 compounds were detected in both SPI and HMMA, 18 of which were hydrocarbon compounds. However, there were significant differences in the content of these common compounds. Maltol content in SPI was merely 21.62 μg/kg, whereas following extrusion, its concentration in HMMA surged over 195-times to 4235.27 μg/kg. The content of 2-furanmethanol in HMMA was 1663.15 μg/kg, while it was not detected in SPI. Similarly, compounds such as furaneol and ethylpyrazine were absent in SPI. These compounds likely formed through Maillard reactions between proteins and residual carbohydrates in SPI under high temperature, high pressure conditions during extrusion. Three off-flavor compounds including hexanal, nonanal, and acetic acid were detected in SPI. Among these, hexanal—the primary contributor to beany flavor—exhibited a significant increase from 22.7 μg/kg in SPI to 577.8 μg/kg in HMMA after extrusion, representing an approximately 25-times increase. This confirms that the degradation of polyunsaturated fatty acids (PUFAs) catalyzed by lipoxygenases (LOX) during extrusion is the primary cause of beany flavor formation (Wang et al., 2021). Nonanal was not detected in HMMA, likely due to its conversion into other compounds through chemical reactions during extrusion. In summary, the flavor profile of SPI was relatively simple, mainly consisting of basic lipid oxidation products. Following high-moisture extrusion processing, the flavor profile of HMMA underwent a fundamental transformation. On the one hand, the Maillard reaction generated a substantial quantity of new heterocyclic compounds contributing positive flavor notes. On the other hand, lipid oxidation reactions were intensified, leading to a significant increase in undesirable compounds such as hexanal. The majority of key flavor compounds identified in HMMA within this study were process generated flavor compounds, rather than those inherent to the raw materials. This contrast clearly demonstrated that the extrusion process was not only pivotal in forming HMMA's texture, but also the decisive factor in creating its unique flavor profile.

### 3.5 Identification of key flavor compounds in HMMA

#### 3.5.1 Aroma-active compounds in HMMA

HMMA with a moisture content of 70% and an extrusion temperature of 160 °C strikes a balance between flavor and texture, making it the chosen sample for this study to further investigate the effects of the extrusion process on odor-active compounds in HMMA. A total of 36 compounds with FD factors ranging from 1 to 8192 were identified in HMMA using GC-O combined with the AEDA method (Table 4). The contribution of these compounds to the aroma of HMMA depended on their FD factors, where higher FD values indicated a more significant impact on the overall aroma. Twenty aroma-active compounds in HMMA had FD values of 16 or higher. Among them, furaneol had the highest FD value of 8192, suggesting that it played crucial roles in the overall flavor profile of HMMA, especially in contributing to the sweet aroma. (+/−)-3-Hydroxy-γ-butyrolactone (FD = 2048) and maltol (FD = 1024) also exhibited high FD values, strongly influencing the sweet aroma, while ethylpyrazine (FD = 2048) exhibited cereal aroma for HMMA. This also indicates that HMMA exhibits pronounced sweet and cereal aromas, consistent with the results shown in Fig. 1 A and 3 A. In addition, AEDA results indicated that hexanal (grass, FD = 256) was the most prominent off-flavor compound in HMMA and also the most abundant one, confirming hexanal as the most significant off-flavor compound in HMMA. 1-octen-3-ol (mushroom, FD = 128) and (E,E)-2,4-decadienal (fat, FD = 16) also exhibited high FD values, indicating their odor characteristics are prominent within the overall aroma profile and significantly influence HMMA's flavor.

**Table 4:** Aroma-active compounds determined by AEDA in HMMA.

No.	Compounds	Odor description	FD
1	Furaneol	Caramel	8192
2	Ethylpyrazine	Roasty,cereal	2048
3	(+/−)-3-Hydroxy-γ-butyrolactone	Sweet, milky	2048
4	Maltol	Caramel	1024
5	2-Methoxy-4-vinylphenol	Medicine, bitter	512
6	Hexanal	Grass	256
7	(5-Methyl-2-furyl)methanol	Fruit, sweet	256
8	1-Octen-3-ol	Mushroom	128
9	2-Ethylhexanol	Floral	128
10	1,2,4,5-Tetramethylbenzene	Sauce	128
11	γ-Butyrolactone	Caramel, sweet	64
12	2(5H)-Furanone	Cereal	64
13	3-Hydroxy-3-methyl-2-butanone	Astringent	32
14	γ-Nonanolactone	Coconut	32
15	Decanoic acid	Rancid	32
16	Hydroxyacetone	Caramel	16
17	(E,E)-2,4-Decadienal	Fat	16
18	Phenylethyl alcohol	Honey, milky	16
19	2-Furylhydroxymethylketone	Metallic	16
20	p-Cresol	Medicine, smoke	16
21	2-Acetylfuran	Balsamic	8
22	2,3-Dihydro-3,5-dihydroxy-6-methyl-4(H)-pyran-4-one	Burnt	4
23	4-Ethenyl-2,6-dimethoxyphenol	Leathery, milky	2
24	Apocynin	Milky, sweet	2
25	Octanoic acid	Sweat	2
26	1-Hydroxy-2-butanone	Burnt, sweet	1
27	3,5-Octadien-2-one	Grass	1
28	Methyl cyclopentenolone	Medicine, bitter	1
29	2-Furanmethanol	Sweat,burnt	1
30	5-Ethyldihydro-2(3H)-furanone	Sweet, milky	1
31	Hexanoic acid	Sweat, sour	1
32	(E)-2-Octenoicacid	Burnt, smoke	1
33	Vanillin	Vanilla	1
34	2-Pentylfuran	Sweet, grass	1
35	2-Methylnaphthalene	Floral	1
36	3-Ethyl-2-hydroxy-2-cyclopenten-1-one	Caramel	1

#### 3.5.2 Quantitation of aroma-active compounds and OAVs

In order to simultaneously monitor the effects of off-flavor compounds on HMMA flavor, off-flavor compounds not detected in GC-O were also quantified (Table 5). Quantification of detected aroma-active compounds revealed that 14 compounds were above their reported odor thresholds. Odor activity values (OAV) were calculated by dividing compound concentrations by their respective thresholds and were found to be highest for hexanal (grass, OAV 3144), 1-octen-3-ol (mushroom, OAV 52) and (E,E)-2,4-decadienal (fat, OAV 52) in general agreement with their high FD factors (FD 16–256). The OAV values of these three odor compounds are significantly higher than those of other flavor compounds in HMMA, indicating that these three compounds have a major impact on the flavor of HMMA. 2-Pentylfuran (Sweet, grass, OAV 25) has an FD value of only 1, while valeraldehyde (almond, OAV 10), and 2-heptanone (soap, OAV 2) were not detected in GC-O. This may be due to low compound concentrations, detectable only in GC–MS but not perceivable in GC-O analysis. This indicated their relatively low direct contribution to the overall aroma profile. However, their larger OAV values suggested they possess potentially significant flavor chemistry attributes. Additionally, 2-methylnaphthalene (floral, OAV 14), maltol (caramel, OAV 13), and furfural (sweat, burnt, OAV 7), which exhibited higher OAV values also significantly influenced the flavor of HMMA. It was also indicated that in GC-O analysis, although 2-methylnaphthalene, 2-furanmethanol, hexanoic acid, and vanillin were only detected in undiluted samples, OAV analysis revealed their substantial influence on HMMA flavor. This demonstrates certain discrepancies between GC-O and OAV analyses in identifying key compounds, underscoring the necessity of employing multiple methodologies for flavor characterization.

**Table 5:** Quantitative results, Odor threshold, and OAVs of key flavor substances in HMMA.

No.	Compounds	Standard Curves	R^2^	Concentrations (μg/kg)	Odorthreshold (μg/kg) a	OAV
1	Furaneol	y = 0.2749× +0.0002	0.9953	67.58 ± 2.03	25	3
2	Ethylpyrazine	y = 1.5298× −0.00009	0.999	2.99 ± 0.096	4000	<1
3	(+/−)-3-hydroxy-γ-butyrolactone	y = 0.2003× + 0.023	0.9953	638.25 ± 55.21	/	/
4	Maltol	y = 0.3482× + 0.4665	0.9991	16,525.44 ± 756.29	1240	13
5	2-Methoxy-4-vinylphenol	y = 1.0067× −0.0023	0.9979	63.47 ± 0.77	19	3
6	Hexanal	y = 0.0475× +0.0269	0.9996	14,147.30 ± 482.40	4.5	3144
7	(5-Methyl-2-furyl)methanol	y = 0.4844× −0.0001	0.9992	37.35 ± 0.45	/	/
8	2-Ethylhexanol	y = 0.8503× +0.0008	0.9993	24.73 ± 0.35	300	<1
9	1-Octen-3-ol	y = 0.7064× −0.00001	0.9995	77.70 ± 1.22	1.5	52
10	1,2,4,5-Tetramethylbenzene	y = 3.0511× - 0.0002	0.9997	2.77 ± 0.13	/	/
11	γ-Butyrolactone	y = 0.3335× +0.0008	0.9989	309.9 ± 4.43	20,000	<1
12	2(5H)-Furanone	y = 0.1987× +0.0134	0.9982	719.80 ± 21.36	/	/
13	3-Hydroxy-3-methyl-2-butanone	y = 0.8989× −0.00005	0.9997	7.46 ± 0.09	/	/
14	γ-Nonanolactone	y = 0.8761× +0.0024	0.9973	35.75 ± 1.08	25	1
15	Decanoic acid	y = 0.3559× −0.0021	0.9914	57.08 ± 6.32	130	<1
16	Hydroxyacetone	y = 0.3006× +0.0099	0.9983	930.80 ± 35.07	10,000	<1
17	Phenylethyl alcohol	y = 1.4152× +0.0011	0.9964	16.29 ± 0.23	3.17	5
18	p-Cresol	y = 1.0013× +0.0011	0.9972	7.21 ± 0.68	2.7	3
19	2-Furylhydroxymethylketone	y = 0.617× +0.00005	0.9976	59.16 ± 1.06	/	/
20	(E,E)-2,4-Decadienal	y = 1.1778× −0.0002	0.9986	5.18 ± 0.11	0.1	52
21	2-Acetylfuran	y = 1.1823× +0.0006	0.999	14.25 ± 0.36	10,000	<1
22	2,3-Dihydro-3,5-dihydroxy-6-methyl-4(H)-pyran-4-one	y = 0.6732× - 0.0088	0.9984	271.07 ± 10.10	35,000	<1
23	4-Ethenyl-2,6-dimethoxyphenol	y = 1.2947× - 0.0011	0.9996	10.21 ± 0.92	/	/
24	Octanoic acid	y = 0.6255× + 0.0034	0.9966	168.92 ± 1.80	1405	<1
25	Apocynin	y = 1.2445× - 0.00008	0.9994	8.78 ± 0.56	780	<1
26	1-Hydroxy-2-butanone	y = 0.2856× - 0.0002	0.9997	24.36 ± 2.17	/	/
27	3,5-Octadien-2-one	y = 0.9454× - 0.0009	0.9998	29.02 ± 0.17	100	<1
28	5-Ethyldihydro-2(3H)-furanone	y = 0.6422× + 0.0001	0.9991	22.43 ± 0.13	260	<1
29	Methyl cyclopentenolone	y = 0.72× - 0.0008	0.9993	15.42 ± 0.66	300	<1
30	2-Furanmethanol	y = 0.1652× + 0.4182	0.9972	13,036.63 ± 394.26	2000	7
31	Vanillin	y = 1.1281× + 0.0059	0.9986	278.91 ± 5.03	64	4
32	Hexanoic acid	y = 0.646× - 0.002	0.9989	142.04 ± 5.05	36	4
33	(E)-2-Octenoicacid	y = 0.4694× - 0.0029	0.9993	27.44 ± 1.60	/	/
34	2-Pentylfuran	y = 1.3222× - 0.0113	0.9997	149.03 ± 3.74	6	25
35	2-Methylnaphthalene	y = 0.0478× - 0.0001	0.9988	135.41 ± 8.22	10	14
36	3-Ethyl-2-hydroxy-2-cyclopenten-1-one	y = 0.7442× - 0.001	0.9995	10.67 ± 0.42	/	/
37	2-Heptanone	y = 1.066× - 0.0092	0.9995	243.71 ± 7.29	140	2
38	1-Hexanol	y = 0.8024× - 0.001	0.9999	40.24 ± 1.28	500	<1
39	1-Pentanol	y = 0.5285× - 0.0016	0.9995	128.62 ± 1.93	4000	<1
40	Valeraldehyde	y = 0.5812× + 0.0037	0.9985	114.72 ± 6.54	12	10
41	Benzaldehyde	y = 1.1577× + 0.0063	0.9983	94.73 ± 1.33	350	<1

a Odor thresholds in water were from reference (Van Gemert, 2018). “/” indicates not found in the literature.

#### 3.5.3 Aroma recombination and omission experiments

**Fig. 6:**
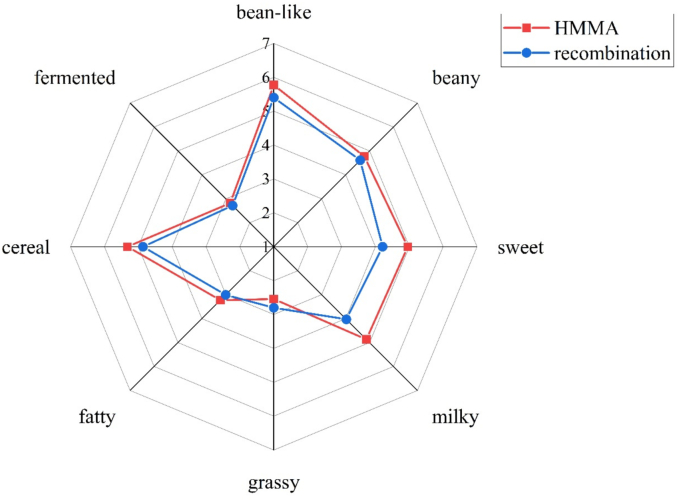
Aroma profiles of HMMA compared with recombination.

An aroma recombination model that consisted of 41 quantitative compounds was evaluated to validated that the compounds identified adequately characterized the HMMA aroma. The aroma recombination model was compared to the original HMMA by using sensory evaluation and the 8 descriptors representing the characteristic odor notes perceivable in HMMA aroma. The results are shown in Fig. 6. The descriptive sensory analysis panel rated the HMMA sample and aroma recombination model similarly, with no significant differences in intensities (*p* > 0.05) for the 8 evaluated attributes. These results suggest that the key aroma compounds in the HMMA were adequately identified and quantified.

In order to evaluate the aroma contribution of individual aroma-active compounds to the overall aroma, 41 omission models were carried out (Table 6), in which a single compound was omitted and evaluated by triangle tests. As shown in Table 6, there were significant differences (*p* < 0.05) in the absence of six compounds. The omission of hexanal, hydroxyacetone, and 5-ethyldihydro-2(3H)-furanone caused highly significant aroma changes (*p* < 0.001), and these 3 compounds contributed grassy, sweet, and milky aroma, which were the main contributors to the aroma profile of the HMMA. When 2-heptanone and 2,3-dihydro-3,5-dihydroxy-6-methyl-4(H)-pyran-4-one were absent, significant differences were observed (*p* < 0.01), and when vanillin was absent, significant difference was observed (p < 0.05), indicating that these compounds contributed to the overall aroma of HMMA. Although 2-heptanone was not detected in GC-O, both OAV calculations and deletion experiments indicated its substantial influence on HMMA's flavor profile. Furaneol and Maltol exhibited high FD factors in Table 4 (8192 and 1024 respectively), yet showed no significant effect in the omission experiments. This may be attributed to masking or synergistic interactions with other compounds, resulting in their individual contributions being less pronounced. In summary, the omission experiments further confirmed that these six odorants were the most key aroma compounds.

**Table 6:** Omission experiments of HMMA.

No.	Compounds	Significance a
1	Furaneol	–
2	Ethylpyrazine	–
3	(+/−)-3-Hydroxy-γ-butyrolactone	–
4	Maltol	–
5	2-Methoxy-4-vinylphenol	–
6	Hexanal	***
7	(5-Methyl-2-furyl)methanol	–
8	2-Ethylhexanol	–
9	1-Octen-3-ol	–
10	1,2,4,5-Tetramethylbenzene	–
11	γ-Butyrolactone	–
12	2(5H)-Furanone	–
13	3-Hydroxy-3-methyl-2-butanone	–
14	γ-Nonanolactone	–
15	Decanoic acid	–
16	Hydroxyacetone	***
17	Phenylethyl alcohol	–
18	p-Cresol	–
19	2-Furylhydroxymethylketone	–
20	(E,E)-2,4-Decadienal	–
21	2-Acetylfuran	–
22	2,3-Dihydro-3,5-dihydroxy-6-methyl-4(H)-pyran-4-one	**
23	4-Ethenyl-2,6-dimethoxyphenol	–
24	Octanoic acid	–
25	Apocynin	–
26	1-Hydroxy-2-butanone	–
27	3,5-Octadien-2-one	–
28	5-Ethyldihydro-2(3H)-furanone	***
29	Methyl cyclopentenolone	–
30	2-Furanmethanol	–
31	Vanillin	*
32	Hexanoic acid	–
33	(E)-2-Octenoicacid	–
34	2-Pentylfuran	–
35	2-Methylnaphthalene	–
36	3-Ethyl-2-hydroxy-2-cyclopenten-1-one	–
37	2-Heptanone	**
38	1-Hexanol	–
39	1-Pentanol	–
40	Valeraldehyde	–
41	Benzaldehyde	–

a * Significant (p ≤ 0.05); ** Highly significant (p ≤ 0.01); *** Very highly significant (p ≤ 0.001).

## 4 Conclusions

This study systematically clarified the regulatory mechanisms of high-moisture extrusion processing on the flavor and texture of soybean HMMA. It was determined that the optimal process conditions were 70% moisture content and 160 °C extrusion temperature, achieving effective suppression of off-flavor and synergistic optimization of the fibrous structure. More importantly, this study comprehensively characterized the key flavor profile of soybean HMMA for the first time. It revealed that characteristic flavor compounds primarily originate from the extrusion process and precisely identified 20 key flavor compounds through molecular sensory science. Among these, furaneol (FD = 8192) was confirmed as the primary source of sweet aroma, while hexanal (OAV = 3144) was identified as the most critical off-flavor compound. Further deficiency experiments indicated that six compounds including hexanal, hydroxyacetone, and 2-heptanone were the core components influencing overall flavor sensory perception. This study elucidates the flavor formation mechanism of HMMA, laying a solid scientific foundation for its precise flavor design and quality enhancement.

## CRediT authorship contribution statement

**Shuwei Wang:** Writing – original draft, Methodology, Investigation, Data curation. **Tianyu Dong:** Methodology, Investigation. **Jie Sun:** Software, Data curation, Conceptualization. **Haitao Chen:** Writing – review & editing, Methodology, Conceptualization. **Huiying Zhang:** Resources, Project administration, Conceptualization.

## Ethical statement

The authors ensure that the work described has been carried out in accordance with The Code of Ethics of the World Medical Association (Declaration of Helsinki) for experiments involving humans.

Ethical approval for sensory evaluation was required by Chinese law. Our study was approved by the Scientific Research Ethics Committee of Beijing Technology and Business University. All experienced panelists were available for sensory evaluation.

The authors confirm that the appropriate protocols for protecting the rights and privacy of all participants were utilized during the execution of the research, including no coercion to participate, full disclosure of study requirements and risks, verbal consent of participants, no release of participant data without their knowledge, and the ability to withdraw from the study at any time.

## Declaration of competing interest

The authors declare that they have no known competing financial interests or personal relationships that could have appeared to influence the work reported in this paper.

## Data Availability

Data will be made available on request.
