# Supplementary material for: Effects of extrusion technology on flavor, texture and microstructure of soy-based high-moisture meat analogs and identification of characteristic aroma components
Source: Food Chem X. 2026 Apr 9;35:103851. doi: 10.1016/j.fochx.2026.103851 (PMC13092859; doi:10.1016/j.fochx.2026.103851)
Supplement: Supplementary file 1 — Supplementary material [file mmc1.docx]

Table S1. Texture characteristics of HMMAs at different moisture contents

|  | Hardness（N） | Adhesiveness （N.mm） | Springiness （mm） | Gumminess （N） | Chewiness （mj） |
| --- | --- | --- | --- | --- | --- |
| HMMA-40% | 11.7±0.72 ^a^ | 0.059±0.008 ^a^ | 2.946±0.1 ^bc^ | 8.137±0.481 ^a^ | 23.95±1.259 ^a^ |
| HMMA-50% | 9.853±0.679 ^b^ | 0.058±0.01 ^a^ | 2.866±0.1 ^c^ | 7.074±0.222 ^b^ | 20.261±0.59 ^b^ |
| HMMA-60% | 9.308±0.462 ^b^ | 0.06±0.005 ^a^ | 3.157±0.127 ^a^ | 6.718±0.224 ^b^ | 21.196±0.557 ^b^ |
| HMMA-70% | 7.233±0.405 ^c^ | 0.033±0.003 ^b^ | 2.724±0.124 ^d^ | 5.326±0.26 ^c^ | 14.494±0.687 ^d^ |
| HMMA-80% | 7.724±0.317 ^c^ | 0.035±0.004 ^b^ | 3.047±0.072 ^ab^ | 5.492±0.238 ^c^ | 16.725±0.488 ^c^ |

Note: Different letters in each row indicate significance (*p* < 0.05).

Table S2. Texture characteristics of HMMAs at different extrusion temperature

|  | Hardness（N） | Adhesiveness （N.mm） | Springiness （mm） | Gumminess （N） | Chewiness （mj） |
| --- | --- | --- | --- | --- | --- |
| HMMA-120 ℃ | 6.324±0.213 ^a^ | 0.036±0.003 ^d^ | 3.218±0.17 ^ab^ | 4.532±0.184 ^b^ | 14.582±0.926 ^bc^ |
| HMMA-130 ℃ | 6.258±0.369 ^b^ | 0.056±0.01 ^b^ | 3.091±0.053 ^bc^ | 4.56±0.243 ^b^ | 14.099±0.828 ^c^ |
| HMMA-140 ℃ | 7.398±0.421 ^a^ | 0.053±0.026 ^bc^ | 2.928±0.091 ^d^ | 5.44±0.223 ^a^ | 15.929±0.853 ^ab^ |
| HMMA-150 ℃ | 5.97±0.127 ^b^ | 0.112±0.023 ^a^ | 2.974±0.104 ^cd^ | 4.433±0.067 ^b^ | 13.188±0.541 ^cd^ |
| HMMA-160 ℃ | 6.359±0.879 ^b^ | 0.034±0.006 ^d^ | 2.984±0.112 ^cd^ | 4.553±0.654 ^b^ | 13.612±2.218 ^c^ |
| HMMA-170 ℃ | 7.298±0.667 ^a^ | 0.039±0.007 ^cd^ | 3.086±0.144 ^bc^ | 5.329±0.446 ^a^ | 16.417±1.22 ^a^ |
| HMMA-180 ℃ | 5.074±0.189 ^c^ | 0.035±0.005 ^d^ | 3.250±0.188 ^a^ | 3.626±0.171 ^c^ | 11.774±0.673 ^d^ |

Note: Different letters in each row indicate significance (*p* < 0.05).

Table S3. Volatile compounds of SPI

| Type | No. | CAS | Compounds | RI | Concentration (μg/kg) | Identification |
| --- | --- | --- | --- | --- | --- | --- |
| Aldehydes | 1 | 66-25-1 | Hexanal | 1091 | 7.959±1.085 | MS, RI, S |
|  | 2 | 124-19-6 | 1-Nonanal | 1392 | 21.041±1.519 | MS, RI, S |
| Subtotal |  |  |  |  | 29.000±2.604 |  |
| Alcohols | 3 | 75-85-4 | Amylene hydrate | 1025 | 151.539±19.505 | MS, RI |
|  | 4 | 1569-50-2 | 3-Penten-2-ol | 1176 | 210.26±7.809 | MS, RI, S |
|  | 5 | 104-76-7 | 2-Ethylhexanol | 1490 | 83.128±1.525 | MS, RI, S |
|  | 6 | 78-70-6 | Linalool | 1551 | 8.503±1.138 | MS, RI, S |
|  | 7 | 54446-78-5 | 1-(2-Butoxyethoxy)ethanol | 1785 | 28.164±1.453 | MS, RI |
| Subtotal |  |  |  |  | 481.594±31.430 |  |
| Acids | 8 | 64-19-7 | Acetic acid | 1464 | 21.697±0.831 | MS, RI, S |
|  | 9 | 79-09-4 | Propanoic acid | 1549 | 19.052±1.117 | MS, RI, S |
|  | 10 | 109-52-4 | Pentanoic acid | 1744 | 15.375±0.829 | MS, RI, S |
|  | 11 | 142-62-1 | Hexanoic acid | 1850 | 43.158±1.446 | MS, RI, S |
|  | 12 | 111-14-8 | Heptanoic acid | 1956 | 9.376±1.859 | MS, RI, S |
|  | 13 | 124-07-2 | Octanoic acid | 2064 | 16.592±2.607 | MS, RI, S |
|  | 14 | 112-05-0 | Nonanoic acid | 2169 | 252.279±37.661 | MS, RI, S |
|  | 15 | 334-48-5 | Decanoic acid | 2275 | 28.533±1.666 | MS, RI, S |
|  | 16 | 65-85-0 | Benzoic acid | 2434 | 25.731±1.642 | MS, RI, S |
| Subtotal |  |  |  |  | 431.793±49.658 |  |
| Esters | 17 | 110-27-0 | Isopropyl myristate | 2034 | 37.614±7.171 | MS, RI, S |
|  | 18 | 131-11-3 | Dimethyl phthalate | 2288 | 17.328±2.893 | MS, RI, S |
|  | 19 | 84-66-2 | Diethyl phthalate | 2362 | 13.595±2.219 | MS, RI |
|  | 20 | 84-69-5 | Diisobutyl phthalate | 2536 | 186.452±32.524 | MS, RI, S |
|  | 21 | 84-74-2 | Dibutyl phthalate | 2691 | 1079.124±206.24 | MS, RI, S |
| Subtotal |  |  |  |  | 1334.113±251.047 |  |
| heterocycles | 22 | 118-71-8 | Maltol | 1954 | 21.618±1.939 | MS, RI, S |
|  | 23 | 872-50-4 | N-Methyl-2-pyrrolidone | 1657 | 1427.733±35.006 | MS, RI, S |
| Subtotal |  |  |  |  | 1449.351±36.945 |  |
| Phenols | 24 | 96-76-4 | 2,4-Di-tert-butylphenol | 2318 | 204.61±39.098 | MS, RI, S |
| Subtotal |  |  |  |  | 204.61±39.098 |  |
| Hydrocarbons | 25 | 6975-98-0 | 2-Methyldecane | 1071 | 31.869±1.369 | MS |
|  | 26 | 1120-21-4 | Undecane | 1109 | 61.8±0.563 | MS, RI, S |
|  | 27 | 100-41-4 | Ethylbenzene | 1130 | 84.906±3.759 | MS, RI, S |
|  | 28 | 1002-43-3 | 3-Methylundecane | 1134 | 8.507±1.157 | MS |
|  | 29 | 138-86-3 | Limonene | 1195 | 1.998±0.136 | MS, RI, S |
|  | 30 | 112-40-3 | Dodecane | 1203 | 23.01±0.16 | MS, RI, S |
|  | 31 | 100-42-5 | Styrene | 1258 | 298.149±2.006 | MS, RI, S |
|  | 32 | 629-50-5 | Tridecane | 1301 | 14.093±0.52 | MS, RI, S |
|  | 33 | 629-59-4 | Tetradecane | 1399 | 15.735±1.069 | MS, RI, S |
|  | 34 | 95-93-2 | 1,2,4,5-Tetramethylbenzene | 1427 | 8.707±0.059 | MS, RI, S |
|  | 35 | 629-62-9 | Pentadecane | 1498 | 7.486±0.44 | MS, RI, S |
|  | 36 | 2882-96-4 | 3-Methylpentadecane | 1567 | 17.039±0.854 | MS |
|  | 37 | 544-76-3 | Hexadecane | 1598 | 24.979±3.06 | MS, RI, S |
|  | 38 | 629-78-7 | Heptadecane | 1698 | 19.45±1.534 | MS, RI, S |
|  | 39 | 593-45-3 | Octadecane | 1797 | 31.407±1.82 | MS, RI, S |
|  | 40 | 629-92-5 | Nonadecane | 1896 | 102.661±4.684 | MS, RI, S |
|  | 41 | 112-95-8 | Eicosane | 1997 | 449.074±64.603 | MS, RI, S |
|  | 42 | 629-94-7 | Heneicosane | 2098 | 624.041±40.919 | MS, RI, S |
|  | 43 | 629-97-0 | Docosane | 2197 | 696.375±65.642 | MS, RI, S |
|  | 44 | 638-67-5 | Tricosane | 2297 | 753.117±78.858 | MS, RI, S |
|  | 45 | 646-31-1 | Tetracosane | 2397 | 860.28±66.026 | MS, RI, S |
|  | 46 | 629-99-2 | Pentacosane | 2497 | 1060.025±110.328 | MS, RI, S |
|  | 47 | 630-01-3 | Hexacosane | 2598 | 1169.354±102.489 | MS, RI, S |
|  | 48 | 593-49-7 | Heptacosane | 2698 | 1004.582±112.22 | MS, RI, S |
|  | 49 | 630-02-4 | Octacosane | 2798 | 886.836±43.947 | MS, RI, S |
| Subtotal |  |  |  |  | 8255.480±708.222 |  |
| Total |  |  |  |  | 12185.941±1119.004 |  |
